# Use of translational fusions to express functional *Klebsiella oxytoca* dinitrogenase reductase in plant mitochondria

**DOI:** 10.1007/s11103-026-01735-5

**Published:** 2026-07-20

**Authors:** Shoko Okada, Xueqin Wang, Christina M. Gregg, Robert S. Allen, Timothy Rhodes, Vanessa Gillespie, Ingrid Venables, Anu Mathew, Jessica K. Bilyj, Keren Byrne, Robert C. DeFeyter, Trevor D. Rapson, Craig C. Wood

**Affiliations:** 1 CSIRO Environment, Acton, ACT 2601 Australia; 2 CSIRO Agriculture and Food, Acton, ACT 2601 Australia; 3 CSIRO Agriculture and Food, St Lucia, QLD 4067 Australia; 4 CSIRO Legal, Acton, ACT 2601 Australia

**Keywords:** NifH, AnfH, Plant mitochondria, *Nicotiana benthamiana*, Solubility, Translational fusion

## Abstract

Engineering crop plants with the biological nitrogen fixation pathway is a longstanding goal of modern agriculture. Dinitrogenase reductase (NifH) is a critical component of the biological nitrogen fixation pathway, with multiple roles in metal cofactor assembly and catalysis. This enzyme must be folded correctly as a soluble homodimer and loaded with the [4Fe-4S] metallocluster for function. Previous studies have found that *Klebsiella oxytoca* (*Ko*) and *Azotobacter vinelandii* (*Av*) NifHs were mostly insoluble when targeted to plant mitochondria. Here we found that a translational fusion of two *Ko*NifH or *Av*NifH monomers, forming *Ko*NifHH or *Av*NifHH synthetic dimers, produced a soluble protein when targeted to plant mitochondria and co-expressed with the putative peptidyl-prolyl cis–trans isomerase NifM. *Ko*NifHH isolated after expression in leaf mitochondria at ambient oxygen showed some acetylene reduction activity, which did not require co-expression of the nitrogenase-specific metallocluster machinery NifS and NifU. This activity increased after iron-sulfur cluster reconstitution in vitro with recombinant NifU. In a parallel study, we tested a translational fusion of a variant iron-only dinitrogenase reductase (*Av*AnfHv6) monomer that was soluble but not active as-isolated from plant mitochondria (*Av*AnfHHv6) (Gregg et al. 2025a). *Av*AnfHHv6 was abundant and fully soluble when isolated from plant mitochondria, like its monomer. *Av*AnfHHv6 was not active as-isolated but could be largely activated by iron-sulfur cluster reconstitution in vitro. This study demonstrates how translational fusions help improve solubility and have the potential to generate an active NifH enzyme within plant mitochondria.

**Supplementary Information:**

The online version contains supplementary material available at 10.1007/s11103-026-01735-5.

## Key message

A translational fusion of two *Klebsiella oxytoca* dinitrogenase reductase monomers was soluble and functional when expressed in *Nicotiana benthamiana* mitochondria, demonstrating its applicability for nitrogen fixation in plants.

**Supplementary Information:**

The online version contains supplementary material available at 10.1007/s11103-026-01735-5.

## Introduction

Modern agricultural systems rely heavily on the use of synthetic nitrogen fertilizer produced by the Haber–Bosch process that was invented over 100 years ago (Smil 1999). This chemical process is extremely energy-demanding, requiring high temperature and pressure to generate ammonia. Furthermore, large amounts of nitrogen fertilizer runoff results in nitrous oxide emissions from the soil and marine eutrophication, which creates a significant environmental cost (Henryson et al. 2020; Ladha et al. 2005). In the last 50 years biological nitrogen fixation (BNF) has been explored as a more economical and environmentally sustainable tool to supply organic nitrogen. BNF is present in a group of microorganisms collectively termed diazotrophs, which use nitrogenase to reduce N_2_ gas to ammonia under ambient temperature and atmospheric pressure. Recent advances in synthetic biology have reignited efforts to introduce nitrogenase into plants, thereby reducing the use of synthetic nitrogen fertilizer.

There are three known nitrogenase systems depending on the unique metal present in the dinitrogenase metal cofactor, namely MoFe (coded by *nif*), FeFe (coded by *anf*) and VFe (coded by *vnf*), nitrogenases. All three systems have two catalytic components, dinitrogenase and dinitrogenase reductase. Dinitrogenase reductases are coded by *nifH*, *anfH,* and *vnfH* for the MoFe, FeFe, and VFe systems, respectively. NifH protein in particular is known to have at least three functions: (1) donation of electrons to dinitrogenase (coded by *nifD* and *nifK*), which reduces N_2_ gas to ammonia, (2) maturation of the P-cluster in NifDK and (3) involvement of FeMo-cofactor assembly by interacting with NifEN (Buren et al. 2020). NifH, VnfH and AnfH are homodimers with a molecular weight of 60–68 kDa and a [4Fe-4S] cluster bridging the two subunits (Georgiadis et al. 1992; Rohde et al. 2018; Trncik et al. 2022). For NifH from *Azotobacter vinelandii* (*Av*) and *Klebsiella oxytoca* (*Ko*) it has been shown that NifM is required for solubility and function (Gavini et al. 2006; Howard et al. 1986; Lei et al. 1999; Yang et al. 2014). NifM is a putative peptidyl-prolyl cis–trans isomerase that is reported to act on either P256 or P258 of *Av*NifH to make it functional (Gavini et al. 2006; Xie et al. 2025). Although NifM is required for *Ko*NifH and *Av*NifH, not all NifH homologues require it for function, including *Av*AnfH (Paya-Tormo et al. 2022; Yang et al. 2014).

Expressing functional nitrogenase in plants requires correctly folded and soluble proteins that are loaded with metalloclusters and protected from oxygen. A possible solution involves targeting of nitrogenase components to the mitochondrial matrix, where respiratory protection permits oxygen-sensitive metalloenzymes to be synthesised and function (Curatti and Rubio 2014). Initial attempts found that *Ko*NifH and *Av*AnfH were mostly insoluble when targeted to the plant mitochondria (Johnston et al. 2023; Okada et al. 2020). To overcome this issue Jiang et al. (2021) identified variants of NifH, often from thermophilic bacteria, which were soluble when targeted to yeast and plant mitochondria. Of the soluble NifH variants, *Hydrogenobacter thermophilus* (*Ht*) NifH isolated from plant mitochondria was partially active for electron donation to *Av*NifDK, and the activity of this enzyme increased when reconstituted with [4Fe-4S] clusters in vitro. *Ht*NifH was even more active when isolated from rice callus, a tissue that cannot generate oxygen from photosynthesis (Baysal et al. 2022; Shim et al. 2020). Recently we published another approach where *Av*AnfH was rationally designed for improved soluble expression in heterologous hosts (Gregg et al. 2025a). One such variant, *Av*AnfHv6, was soluble when expressed in plant mitochondria and was active after [4Fe-4S] cluster reconstitution in vitro.

Our lab has previously shown that the insoluble *Ko*NifD when expressed in *Nicotiana benthamiana* mitochondria could be made soluble by translationally fusing it to its heterodimeric partner NifK (Allen et al. 2020). We reasoned that the physical proximity of NifD and NifK in the plant mitochondrial matrix may have assisted with the interaction of NifD and NifK so that the two subunits could form a soluble heterodimer in the correct conformation. Therefore, we hypothesized that the formation of NifH or AnfH homodimers in plant mitochondria may similarly be improved by translationally fusing two NifH or AnfH monomers. In this study we expressed translational fusions of two *Ko*NifH or *Av*NifH monomers (defined as *Ko*NifHH or *Av*NifHH, respectively) in *N. benthamiana* leaf, which became markedly soluble only when co-expressed with NifM. *Ko*NifHH isolated from *N. benthamiana* leaf mitochondria was active as-isolated for electron donation to *Av*NifDK, which did not require co-expression of *Av*NifS and *Av*NifU. Furthermore, we assessed for activity of the translational fusion of *Av*AnfHv6 (*Av*AnfHHv6) expressed in *N. benthamiana* leaf mitochondria. *Av*AnfHHv6 was not active as-isolated, but it could be activated by in vitro reconstitution of [4Fe-4S] clusters when combined with iron-sulfur cluster-loaded NifU. This study adds to the growing body of evidence that (1) [4Fe-4S] cluster loading, and (2) maintenance of these catalytic components in an active form, are two major bottlenecks for engineering the nitrogen fixation pathway in plants that need to be solved.

## Material and methods

### Expression constructs

All plant expression constructs were assembled using Golden Gate parts and toolkit (Engler et al. 2014), and following the Golden Gate assembly protocol (Weber et al. 2011). Genes coding for two *Ko*NifH or *Av*AnfHv6 monomers were translationally fused together with either an ArsA linker (Yang et al. 2018) or a Twin-Strep-tag® (TS) as the linker for Strep-Tactin® affinity chromatography. Furthermore, a series of termination signals containing the intronless tobacco extensin 3’ UTR, followed by the cauliflower mosaic virus 35S terminator, then the tobacco Rb7 matrix attachment region (MAR) were added at the 3’ end of the coding sequence to enhance expression of the transgene (herein termed TTm) (Diamos and Mason 2018; Diamos et al. 2016; Gregg et al. 2025a). All versions of dinitrogenase reductase proteins were targeted to *N. benthamiana* mitochondria by translationally fusing either the first 51 amino acid residues of the *A. thaliana* γATPase (mitochondrial target peptide (MTP)FAγ) (Okada et al. 2020), which was followed by a hemagglutinin (HA) tag for protein detection, or a CoxIV mitochondrial targeting peptide of the first 29 amino acid residues of the *Saccharomyces cerevisiae* cytochrome c oxidase subunit 4 (Jiang et al. 2021) followed by a Twin-Strep-tag® on the N-terminus of dinitrogenase reductase. Co-expression constructs coding for *Av*NifS and *Av*NifU were targeted to plant mitochondria using the first 31 amino acid residues of *Arabidopsis thaliana* CPN60 (Prasad and Stewart 1992) and the first 70 amino acid residues of the subunit 9 F_0_ATPase from *Neurospora crassa* (SU9) (Buren et al. 2017), respectively. All expression constructs have been assigned a code ‘SN’ followed by a number, which is summarised in Table 1 along with a description of the general design features for each construct.

**Table 1:** Key features of the expression constructs used in this study

Plasmid ID	Expression system (promoter, terminator other than CMV 35S terminator)	Description	MPP-processed MW (kDa)	Unprocessed MW (kDa)
P19	e35S	P19 viral suppressor	n/a	n/a
pRA01	e35S	MTPFAγ^77^::GFP^†^	31.1	35.7
SL133	e35S	CPN60MTP::*Av*NifS, co-expressed with SU9MTP::*Av*NifU	43.8	47.4
SL133	e35S	SU9MTP::*Av*NifU, co-expressed with CPN60MTP::*Av*NifS	33.6	40.7
SN18	e35S	MTPFAγ::*Ko*NifH::HA	34.3	38.8
SN207	e35S	6xHis::*Ko*NifM::HA	n/a	31.8
SN283	e35S	MTPFAγ::*Ko*NifH::ArsAlinker::NifH::HA	68.8	73.3
SN303	e35S	6xHis::*Ko*NifH::ArsAlinker::NifH::HA	n/a	68.7
SN360	e35S	SU9MTP::KoNifM	30.6	37.7
SN604	e35S	MTPFAγ::HA::*Av*NifM	35.3	39.9
SN605	e35S	MTPFAγ::*Av*NifM	34.1	38.7
SN638	e35S	CoxIVMTP::TS::*Ko*NifH::ArsAlinker::NifH	70.8	73.7
SN656	e35S, TTm	MTPFAγ::HA::*Ko*NifH::TS::NifH	69.3	74.1
SN679	e35S, TTm	MTPFAγ::HA::*Av*NifH::ArsAlinker::NifH	68.8	73.6
SN682	e35S, TTm	MTPFAγ::HA::*Av*AnfH::ArsAlinker::AnfH	69.8	65.2
SN740	e35S	6xHis::*Av*NifM	n/a	34.0
SN775	e35S	MTPFAγ::*Av*AnfHv6::TS::AnfHv6	64.2	68.8
SN776	e35S, TTm	MTPFAγ::*Av*AnfHv6::TS::AnfHv6	64.2	68.8
SN807	e35S	alaMTPFAγ::*Ko*NifH::ArsAlinker::NifH::HA	n/a	72.7
SN843	e35S, TTm	alaMTPFAγ::*Av*AnfHv6::TS::AnfHv6	n/a	68.2
SN844	e35S	HA:: *Av*AnfHv6::TS::AnfHv6	n/a	64.4

All SN constructs used cauliflower mosaic virus (CMV) 35S terminator unless otherwise described. MPP, mitochondrial processing peptidase; MW, molecular weight; n/a, not applicable. † For pRA01 the MTPFAγ^77^ that was translationally fused to green fluorescent protein (GFP) was the first 77 amino acids of the *A. thaliana* γATPase. TTm, transcription termination signals containing the intronless tobacco extensin 3’ UTR, followed by the cauliflower mosaic virus 35S terminator, then the tobacco Rb7 matrix attachment region

### *N. benthamiana* leaf transient expression and western blot analysis

*N. benthamiana* leaf transient expression for initial protein expression testing was conducted essentially as per Gregg et al. (2025a). Briefly, *N. benthamiana* were grown in a growth chamber (Conviron) at 24 °C under a 16:8 h light: dark cycle in vermiculite (grade 3, Ausperl) provided with Hyper Grow GP nutrient solution (Hyper Grow, Australia). Leaves of 5–6 week old *N. benthamiana* plants were infiltrated on the abaxial side using a 1 ml syringe with infiltration mixes of *Agrobacterium tumefaciens* strain GV3101 cultures containing expression constructs or viral suppressor p19, at OD_600nm_ = 0.1 for each GV3101 culture. At the end of the third day post-infiltration (dpi, approximately 18 h before harvest), plants were moved into the dark, leaves harvested the next day in the dark, snap-frozen in liquid nitrogen and stored at − 80 °C until use. For large scale expression and purification of *Ko*NifHHv2 or *Av*AnfHHv6, *N. benthamiana* plants were infiltrated either manually by using a syringe according to Allen et al*.* (2020) or by vacuum infiltration. Vacuum infiltration of plants was performed as follows: plants were immersed into infiltration mixes of *A. tumefaciens* GV3101 containing expression constructs or viral suppressor p19, along with silwet L-77 (Phyto Technology Laboratories LLC) at a final concentration of 0.025% of the infiltration mix, by sitting the pot with the plants upside-down in containers filled with infiltration mixes. The vacuum chamber (BVV) was connected to the pump via a small cold-trap chamber and vacuum was applied to 200 mbar. The vacuum was gradually released to infiltrate the plants (typically 45 plants), which were returned to the growth chamber and leaves harvested in the dark at four dpi and stored as described above. Western blot analysis to compare expression of the soluble and insoluble fractions of *Ko*NifHH, *Av*NifHH or *Av*AnfHHv6 in *N. benthamiana* was performed as per Allen et al. (2020) with the following modifications: The solubility buffer was 100 mM Tris–HCl pH 8.6, 200 mM NaCl, 10% v/v glycerol, 2 mM tris(2-carboxyethyl)phosphine (TCEP), 0.2 mM phenylmethylsulphonyl fluoride (PMSF), and 10 μM leupeptin.

### Proteomic analysis

Approximately 100 mg of leaf that was infiltrated with *A. tumefaciens* strain GV3101 cultures containing expression constructs was harvested four dpi and ground in 500 ul solubility buffer. The ground sample was transferred to 1.5 ml microcentrifuge tubes and centrifuged at 21,000 × *g* for 10 min at 4 °C, and supernatant was collected, and protein concentration measured with the bicinchoninic acid (BCA) protein assay kit (Thermo Fisher Scientific) against known concentrations of bovine serum albumin. One hundred (100) ug of supernatant was applied to the 3 kDa filter prior to reduction in UTC buffer (100 mM Tris–HCl pH 8.5, 8 M urea, 2 M thiourea, 4% CHAPS, with 50 mM DTT), alkylation (50 mM iodoacetamide) and tryptic digestion using the standard FASP protocol. Sequencing grade porcine trypsin (Promega, Alexandria, Australia) was added at an enzyme to protein ratio of 1:50 (0.01 µg/µl, i.e. 2 µg in 200 µl of 50 mM ammonium bicarbonate with 1 mM CaCl_2_) to the samples and incubated for 16 h at 37 °C in a thermomixer at 300 rpm. The extracted tryptic peptides were dried and resuspended in 100 µl of 0.1% formic acid (~ 1 µg/µl). Initially, these peptides were further diluted to 0.25 µg/ µl (12.5 ul was added to 37.5 ul of 0.1% formic acid) prior to sequential window acquisition of all theoretical fragment ion spectra (SWATH) mass spectrometry analysis.

Samples were analysed using an ACQUITY UPLC M-Class system (Waters) coupled to a ZenoTOF 7600 mass spectrometer with an Optiflow (1-50 µl) source (SCIEX, USA). Prior to MS analysis, samples were chromatographically separated on a nanoEase™ M/Z HSS T3 column (300 µm × 150 mm, 1.8 µm, 100 Å, Waters) heated to 40 °C, using a flow rate of 5 µl/min where mobile phases A and B were 0.1% formic acid in water and 0.1% formic acid in acetonitrile, respectively. A 45 min gradient was applied (time, % of mobile phase B: 0 min, 3%; 1 min, 3%; 30 min, 35.0%; 37 min 80%; 39 min, 80%; 40 min, 3%; 45 min, 3%). To compensate for any injection volume variance, 2 µl of each sample (~ 500 ng protein) was loaded. TOF MS scans were acquired at 300–1500 m*/z* with an accumulation time of 0.1 s. A SWATH acquisition scheme with 65 variable windows covering a precursor mass range of 300.00–750.00 m/z and 0.1 s accumulation time was used with Zeno pulsing selected. The ion source gas 1 and 2 were set to 20 and 15 psi, respectively; curtain gas to 35, CAD gas to 7, and source temperature to 150 °C; spray voltage was set to 5000 V.

Spectral data were searched against the species-specific *N. tabacum* database appended with cRAP (common repository of adventitious proteins) and custom sequences SN656 (*Ko*NifHH)_SN360 (*Ko*NifM)_SL133 (*Av*NifS_*Av*NifU). This database was obtained from the UniProt Proteome UP000084051. A second database containing the MPP-cleaved custom amino acid sequences was also used to check MTP processing.

### Isolation of *Ko*NifHH or *Av*AnfHHv6 from *N. benthamiana* leaf

Isolation of *Ko*NifHHv2 (SN656) or *Av*AnfHHv6 (SN776) expressed in *N. benthamiana* leaves was conducted in a COY anaerobic chamber with 97–98% N_2_ /2–3% H_2_ atmosphere as follows: typically 150–200 g of frozen leaf tissue was ground in lysis buffer (100 mM Tris–HCl pH 8.6, 200 mM NaCl, 10% v/v glycerol, 2 mM sodium dithionite (DTH), 0.2 mM PMSF, 10 μM leupeptin) with a NutriBullet blender, filtered through miracloth (Merck Millipore) and centrifuged at 142,400 × *g* for 1 h at 4 °C. The resulting supernatant was filtered through a 0.2 μm PVDF membrane filter and loaded onto a 5 ml Streptactin® XT 4Flow® column (IBA Life Sciences) at 2.5 ml/min and ambient temperature. After supernatant loading, the column was washed with 10 column volumes of wash buffer (100 mM Tris–HCl pH 8.6, 200 mM NaCl, 10% v/v glycerol, 2 mM DTH), after which the bound protein was eluted with wash buffer containing 50 mM biotin. The eluate was concentrated down to approximately 300 μl with an Amicon 50 kDa cut-off centrifugal filter unit (Merck Millipore) and stored under liquid nitrogen until use. Densitometry of the Coomassie stain of the sodium dodecyl-sulfate polyacrylamide gel electrophoresis (SDS-PAGE) was conducted to determine the proportion of the isolated target protein in the concentrated eluate with the analysis software in the Amersham Imager 600 (GE Healthcare).

### Acetylene reduction assay

Prior to iron-sulfur cluster reconstitution and acetylene reduction assay (ARA), the elution buffer of isolated *Ko*NifHH or *Av*AnfHHv6 was replaced with 50 mM Tris, 100 mM NaCl, pH 7.7, or 2 mM ATP, 5 mM MgCl_2_ in 25 mM MOPS buffer, pH 7.5, respectively, using the 50 or 30 kDa cut-off centrifugal filter unit, respectively. Protein concentrations of the purified protein after buffer replacement were measured with the BCA protein assay kit (Thermo Fisher Scientific) against known concentrations of bovine serum albumin. ARA was performed according to Jiang et al*.* (2021) with the following modifications: The final volume of each reaction was 370 μl (containing 2 mM ATP, 5 mM MgCl_2_, 7.5 mM phosphocreatine, 100 µg/ml creatine phosphokinase, 3 mM DTH in 37.5 mM Tris–HCl pH 7.9 and 50 mM NaCl for *Ko*NifHH, or 25 mM MOPS buffer pH 7.5 for *Av*AnfHHv6, and NifDK isolated from *A. vinelandii* DJ2102) (Gregg et al. 2025a), which was assembled into a 3.7 ml Exetainer vial (Labco UK). Three hundred and seventy (370) µl (vol.) for *Ko*NifHHv2 or 1 ml (vol.) for *Av*AnfHHv6 of acetylene gas was injected into the vials, and the reaction mix was incubated at 30 °C for 30 min. The acetylene gas used for the assay was exposed to 10 mM DTH in 100 mM Tris pH 8 to remove any traces of oxygen before use. The reaction was then terminated by adding 100 µl of 8 M NaOH and production of ethylene was measured by gas chromatography with flame ionization detection (Allen et al. 2020). Positive controls were conducted with *Av*NifH or *Av*AnfH isolated from *A. vinelandii* DJ2102 or DJ2241, respectively (Gregg et al. 2025a), and *Av*NifDK at a ratio of 40:1 for *Av*NifH:*Av*NifDK and 20:1 for *Av*AnfH:*Av*NifDK. When setting up the acetylene reduction assay, the proportion of *Ko*NiHHv2 or *Av*AnfHHv6 were taken into account when calculating the volume required to have the NifH/AnfH:*Av*NifDK ratio consistent between replicates and in line with the positive control reactions. Preparation of apo-*Av*NifH, isolation of *Av*NifU expressed in *E. coli* (*Ec-Av*NifU) and iron-sulfur cluster reconstitution of *Ko*NifHHv2, *Av*AnfHHv6 and apo-*Av*NifH is described in (Gregg et al. 2025a). In short, *Av*NifU with a Twin-Strep-tag® at the N-terminus was expressed in *E. coli* BL21 (DE3) and purified under anaerobic conditions. After purification, *Ec-Av*NifU was chemically reconstituted with 2.5 mM (NH_4_)_2_Fe(SO_4_)_2_ and 0.5 mM Na_2_S to increase iron-sulfur cluster loading on *Ec-Av*NifU. The mixture was incubated for at least 16 h, after which the buffer was replaced with Mg-ATP buffer (10 mM MgCl_2_, 2 mM ATP, 25 mM Tris pH 7.4) to remove excess Fe^2+^ and S^2−^ using a MiniTrap G25 column. This chemically reconstituted *Ec-Av*NifU was then used for in vitro reconstitution of NifH and AnfH proteins. Prior to iron-sulfur cluster reconstitution, the elution buffer was exchanged as described for the ARA assays above for *Av*AnfHHv6, and for *Ko*NifHHv2 the exchange buffer was 2 mM ATP, 5 mM MgCl_2_ in 50 mM Tris, 100 mM NaCl, pH 7.7. The ratio of *Ec-Av*NifU:*Ko*NifHHv2 or *Ec-Av*NifU:*Av*AnfHHv6 was 2:1 and the mixture was incubated for 30 min or overnight, respectively. 

### Testing diazotrophic growth of *K. oxytoca* nif system with *Ko*NifHHv2 in *E. coli*

A Golden Gate-compatible variant of pKU7017 (Wang et al. 2013) was generated to test whether *Ko*NifHHv2 could functionally replace native *Ko*NifH in the full *K. oxytoca nif* operon. The construct contained the complete *K. oxytoca nif* gene cluster (*nifJHDKTYENXUSVWZMFBQLA*) with a green fluorescent protein (GFP) dropout cassette inserted at the *nifH* position of the pKU7017 *Klebsiella oxytoca nif* operon to enable simple replacement of *nifH* variants. The GFP dropout cassette was first assembled in pTU1-A6 using the constitutive promoter J23100, the GFP coding sequence, and the BBa_B0015 terminator from the EcoFlex MoClo kit (Moore et al. 2016). The entire promoter-GFP-terminator cassette was amplified using primers binding to the 5’ end of the J23100 promoter and the 3’ end of the BBa_B0015 terminator. These primers introduced outward-facing BsaI sites and inward-facing BbsI sites with defined Golden Gate overhangs compatible with the *nif* operon assembly. For assembly of the modified *nif* operon, it was divided into four fragments for cloning into the pAGM4723 Golden Gate acceptor vector using BbsI (Weber et al. 2011). The first fragment was amplified by PCR from the upstream region of *nifJ* to the start of the initiating methionine of *nifH*. The GFP dropout cassette was inserted at this *nifH* position. The remaining fragments comprised the downstream *nif* genes, including *nifDKTYENX*, *nifUSVWZM*, and *nifFBQLA*. After the initial Golden Gate assembly using BbsI, the internal BsaI sites flanking the GFP dropout cassette were retained and designed such that replacement of the cassette with *nifH*-derived inserts were precisely in-frame. This enabled scarless insertion of wildtype *KonifH*, *KonifHHv2*, or stop-codon-disrupted *KonifH*, with the initiating methionine and terminal stop codon positioned identically to the native *nifH* coding sequence. Correct plasmids were identified by sequencing prior to function testing in *E. coli*. *E. coli* BL21 Star cells were transformed with plasmids by electroporation and selected on Luria–Bertani (LB) agar containing 25 µg/ml chloramphenicol. Single colonies were inoculated into 2 ml LB medium with 25 µg/ml chloramphenicol and grown at 37 °C with shaking at 220 rpm until cultures were just visibly turbid. The final OD_600nm_ was typically between 0.1 and 1.0 before being placed on ice. Once all cultures had been collected, and after the final culture had spent 15 min on ice, cells were harvested by centrifugation at 1300 × *g* for 1.5 min at room temperature and supernatants discarded. Cell pellets were washed twice with sterile 0.85% NaCl, with centrifugation at 1300 × *g* for 1.5 min between washes. Washed cells were resuspended in sterile 0.85% NaCl and adjusted to OD_600nm_ = 0.1. Standardized suspensions were then diluted by adding 2 µl of cells to 998 µl nitrogen-free M9 medium to give a final OD_600nm_ of 2 × 10⁻^4^. For each strain 20 µl of the diluted suspension was spotted onto nitrogen-free M9-Phytagel plates. The base medium for these plates contained 6.0 g/l Na₂HPO_4_, 3.0 g/l KH_2_PO_4_, 1.0 g/l NaCl and 25 mM MOPS, pH 7.4 with NaOH. The medium was supplemented with 5 mM sodium fumarate and solidified with 0.20% Phytagel. After autoclaving, the M9-Phytagel medium was cooled to 65 °C before addition of filter-sterilised components. Final concentrations of components were 0.8% (w/v) glucose, 100 µM CaCl_2_, 1 mM MgSO_4_, 100 µM ferric citrate, 40 µM ZnSO_4_, 1 µM Na_2_MoO_4_, 1 µM MnSO_4_, 20 µM biotin, 1.5 µM thiamine hydrochloride and 25 µg/ml chloramphenicol. The medium was mixed and poured into 90 mm Petri dishes at 40 ml per plate, and dried for at least 1 h. LB agar containing appropriate antibiotic were used for permissive growth and dilution control. Plates were incubated anaerobically for seven days and diazotrophic growth was visually assessed.

## Results

### A translational fusion of two *Ko*NifH monomers is soluble when co-expressed with *Ko*NifM and targeted to plant mitochondria

**Fig. 1:**
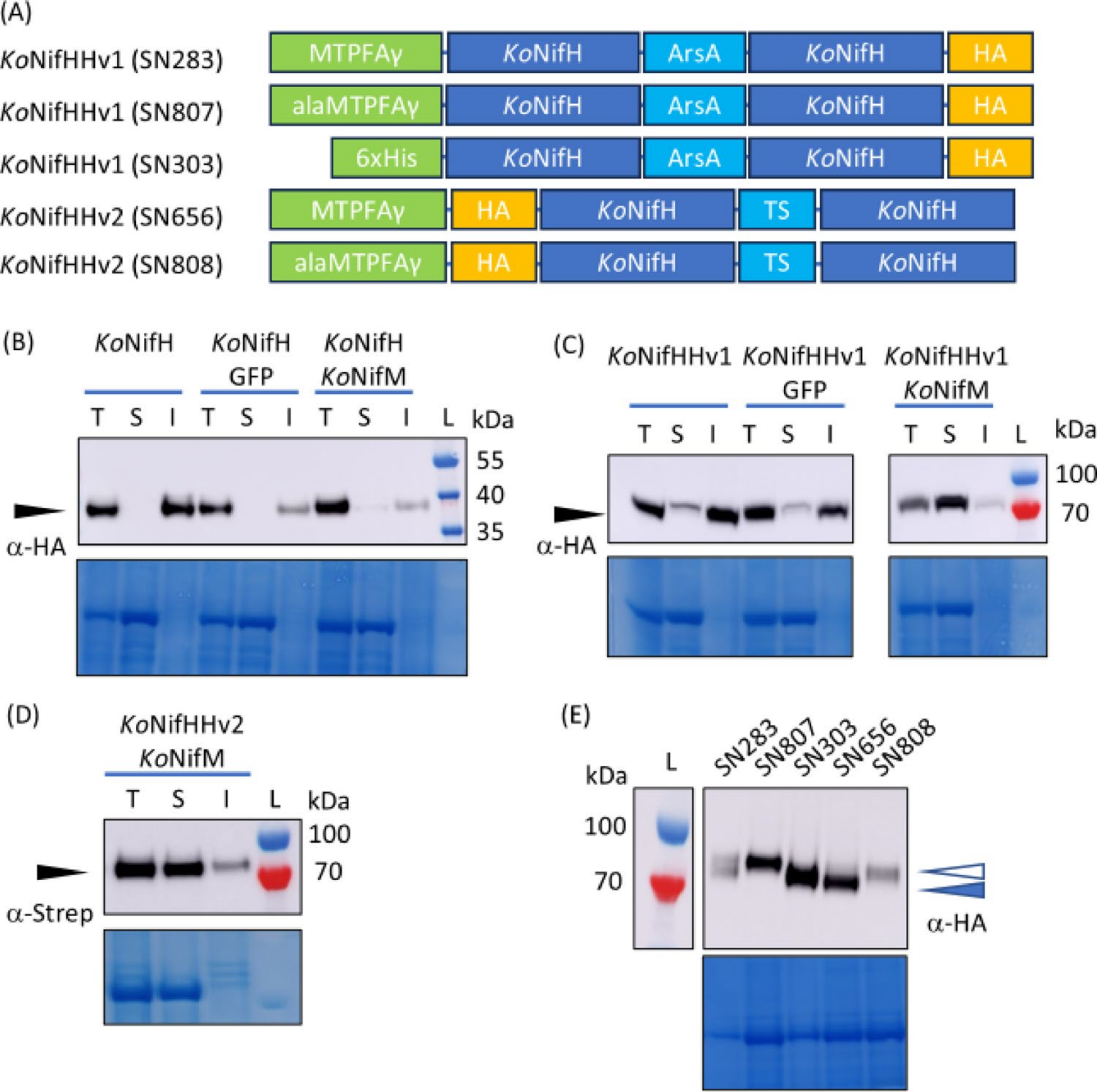
Assessing solubility and processing of *Ko*NifH monomer or translationally fused NifH dimers (*Ko*NifHH) targeted to *N. benthamiana* mitochondria. **A** Configuration of the *Ko*NifHHv1 (linked with ArsA linker) and *Ko*NifHHv2 (linked with Twin-Strep-tag®) translational fusions used for this analysis (MTPFAγ, first 51 amino acid residues of *Arabidopsis thaliana* γATPase preprotein; ArsA, ArsA linker; HA, hemagglutinin tag; TS, Twin-Strep-tag®; genetic parts in diagram are not to scale); **B** Anti-HA western blot (top) and corresponding Coomassie-stained SDS-PAGE (bottom) of total (T), soluble (S), and insoluble (I) fractions of *Ko*NifH (SN18) transiently expressed in *N. benthamiana* leaf, with or without green fluorescent protein (GFP, pRA01) or *Ko*NifM (SN360); **C** Anti-HA western blot (top) and corresponding Coomassie-stained SDS-PAGE (bottom) of total (T), soluble (S), and insoluble (I) fractions of *Ko*NifHHv1 (SN283) transiently expressed in *N. benthamiana* leaf mitochondria, co-expressed with or without green fluorescent protein (GFP, pRA01) or *Ko*NifM (SN360). Black triangles point to *Ko*NifH in (**B**) and *Ko*NifHHv1 in (**C**); **D** Anti-strep western blot (top) and corresponding Coomassie-stained SDS-PAGE (bottom) of total (T), soluble (S), and insoluble (I) fractions of *Ko*NifHHv2 (SN656), black triangle points to *Ko*NifHHv2; **E** Anti-HA western blot (top) and corresponding Coomassie-stained SDS-PAGE (bottom) of different forms of *Ko*NifHHv1 and v2 shown schematically in (**A**), transiently expressed in *N. benthamiana* leaf mitochondria to show processing of the mitochondrial targeting peptide of *Ko*NifHHv1 and v2 by the mitochondrial processing peptidase (MPP). Total fractions were used for this analysis. All forms of *Ko*NifHH were co-expressed with *Ko*NifM (SN360). Open triangle denotes signals corresponding to unprocessed *Ko*NifHHv1 (SN807) and v2 (SN808), solid blue triangle denotes signals corresponding to MPP-processed *Ko*NifHHv1 (SN283) and v2 (SN656). Note that *Ko*NifHHv1 (SN283) also has a slower migrating signal in line with unprocessed *Ko*NifHHv1 (SN807. L, protein ladder. Full-length blot and gel images are shown in Supplementary Fig. 1 for (B), Supplementary Fig. 3 for (C), Supplementary Fig. 5 for (D), and Supplementary Fig. 6 for (E). Details of constructs are summarised in Table 1

As reported in Okada et al*.* (2020), monomeric *Klebsiella oxytoca* NifH (*Ko*NifH) protein translationally fused to the 51 amino acid mitochondrial targeting peptide (MTP) of the *Arabidopsis thaliana* ATPase gamma subunit (MTPFAγ) at the N-terminus was insoluble when targeted to *N. benthamiana* leaf mitochondria. We sought ways to increase the solubility of *Ko*NifH. Co-expression of monomeric *Ko*NifH with the maturase protein *Ko*NifM (construct SN360, Table 1) within plant mitochondria only marginally increased the solubility of *Ko*NifH (Fig. 1B, Supplementary Fig. 1). The solubility further slightly increased with co-expression of iron-sulfur cluster machinery proteins NifS and NifU from *A. vinelandii* (*Av*NifS and *Av*NifU) (Supplementary Fig. 2). Next, as *Ko*NifH functions as a homodimer, we hypothesised that a translational fusion of the two subunits might assist forming a complex in the correct configuration and increase solubility. A fusion protein (*Ko*NifHHv1, Fig. 1A) was designed having two *Ko*NifH monomers joined with an intervening oligopeptide linker. The initial linker design used a 25-amino acid oligopeptide derived from ArsA that was previously used to dimerize *Ko*NifH and shown to be functional for acetylene reduction when expressed in *Escherichia coli* (Yang et al. 2018). The initial *Ko*NifHHv1 fusion protein, with a C-terminal hemagglutinin (HA) epitope for detection, was targeted to *N. benthamiana* mitochondria using the same MTP as the insoluble monomeric *Ko*NifH and the same CaMV e35S promoter and 35S transcription terminator for expression (Table 1). Mitochondrial *Ko*NifHHv1 expressed on its own was slightly soluble, but in contrast to the monomeric *Ko*NifH, it became mostly soluble when co-expressed with *Ko*NifM (Fig. 1C, Supplementary Fig. 3). When *Ko*NifHHv1 was co-expressed with GFP that was also targeted to leaf mitochondria, solubility was not increased (Fig. 1C, Supplementary Fig. 3), showing that the effect on solubility was specific to *Ko*NifM. The solubility of *Ko*NifHHv1 did not change when *Av*NifS and *Av*NifU were co-expressed along with *Ko*NifM (Supplementary Fig. 2). An expression construct was then designed with a CoxIV MTP followed by a Twin-Strep-tag® (TS) at the *N* terminus of *Ko*NifHHv1 for affinity purification (SN638), herein defined as CoxIV-TS-*Ko*NifHHv1 (Jiang et al. 2021). This CoxIV-TS-*Ko*NifHHv1 fusion protein also became soluble when co-expressed with *Ko*NifM, but not as much as MTPFAγ-*Ko*NifHHv1 (Supplementary Fig. 4).

A second version of the *Ko*NifHH translational fusion polypeptide (*Ko*NifHHv2, Fig. 1A) was made with a Twin-Strep-tag® as the linker as well as to allow for affinity purification. The position of the HA tag was also switched to between MTP and *Ko*NifHHv2 (Fig. 1A). This genetic construct (SN656) was expressed from the same e35S promoter, but with the more efficient hybrid terminator defined as the TTm transcription terminator (Gregg et al. 2025b). As for *Ko*NifHHv1, *Ko*NifHHv2 became mostly soluble when it was co-expressed with *Ko*NifM, indicating that different linkers can be used to fuse the two *Ko*NifH monomers (Fig. 1D, Supplementary Fig. 5).

To show evidence of processing by the mitochondrial processing peptidase (MPP) and thereby mitochondrial targeting of *Ko*NifHHv1 and *Ko*NifHHv2, modified versions were generated as molecular weight controls, one having an inactivated MTP by alanine substitutions (alaMTPFAγ in Fig. 1A) (Allen et al. 2020). A further control replaced the MTP sequence with a 6xHis tag at the N-terminus (Fig. 1A), providing for cytosolic localisation of a protein having about the same electrophoretic mobility as the MPP-processed *Ko*NifHHv1 and *Ko*NifHHv2. Western blot analysis of *Ko*NifHHv1 expressed in *N. benthamiana* leaves showed two signals – one aligned with the cytosolic version and the other migrating slower and aligned with the non-processed alaMTPFAγ version (Fig. 1E, Supplementary Fig. 6). By contrast, *Ko*NifHHv2 generally showed the faster migrating signal, consistent with mitochondrial targeting and efficient cleavage within the MTP sequence by MPP (Fig. 1E, Supplementary Fig. 6). These results, including for solubility, support a conclusion of successful targeting of *Ko*NifHHv1 and *Ko*NifHHv2 to the mitochondrial matrix in a soluble form, albeit with some unprocessed protein also present.

### A translational fusion of two *Av*NifH monomers is also soluble in plant mitochondria

*A. vinelandii* NifH (*Av*NifH) expressed in monomer form was also reported to be insoluble when targeted to plant mitochondria (Jiang et al. 2021). To determine whether the increased solubility of the translationally fused *Ko*NifHH dimers occurred more generally, a translational fusion of two monomers of *Av*NifH with the ArsA linker was constructed (defined as *Av*NifHH, SN679, Table 1), and co-expressed with *A. vinelandii* NifM (*Av*NifM) (N-terminal HA-tagged *Av*NifM, SN604; no-tagged *Av*NifM, SN605, Table 1). When targeted to *N. benthamiana* leaf mitochondria using MTPFAγ and the TTm transcription terminator, *Av*NifHH was partially soluble (Supplementary Fig. 7). However, the soluble fraction of *Av*NifHH was never as abundant as for *Ko*NifHHv2. To investigate if the use of a linker would affect correct folding of NifH we used Alphafold 3 (Abramson et al. 2024) to construct predicted protein structures of *Av*NifHH and *Ko*NifHHv2 and compared them to a reported crystal structure of *Av*NifH (Protein Data Bank accession 2AFH Fe protein chain E) (Supplementary Fig. 8). Pairwise alignment of *Ko*NifHHv2 and *Av*NifHH, and *Av*NifHH and 2AFH showed low root mean square deviation (RMSD, ranging between 0.87 and 1.5 Å) and high template modelling (TM) score (ranging between 0.91 and 0.98), suggesting that the linker did not interfere with the folding of the two connected NifH proteins. Because the abundance of *Av*NifHH expressed in *N. benthamiana* leaf was low, we focussed on *Ko*NifHHv2 for functional analyses described below.

### Cytosolically expressed NifM can solubilize *Ko*NifHH targeted to mitochondria

The NifM proteins in the above experiments were mitochondrially targeted (MTP-NifM). To determine whether NifM proteins could use the NifHH dimers as substrates in the cytosol and still help solubilize NifHH proteins targeted to the mitochondrial matrix, constructs to target NifM proteins to the cytosol were generated. When *Ko*NifHHv2 was co-expressed with a cytosolic *Ko*NifM (SN207, Table 1) in *N. benthamiana* leaves and analysed by western blot, *Ko*NifHHv2 was almost as soluble as when it was co-expressed with *Ko*NifM that was targeted to the mitochondria (comparison between Fig. 1D and Supplementary Fig. 9). Similarly, *Av*NifHH was also more soluble when co-expressed with cytosolically targeted *Av*NifM (SN740, Table 1) than without NifM (comparison between Supplementary Figs. 7 and 9). These results suggest that the NifM protein was acting on *Ko-* and *Av*NifHH before it entered the mitochondrial matrix.

### A translational fusion *Nb-Ko*NifHHv2 co-expressed with *Nb-Ko*NifM is partially functional

*K*oNifHHv2 protein was purified from *N. benthamiana* leaves and assessed for function in vitro. For this, *Ko*NifHHv2 was co-expressed with MTP-*Ko*NifM and either with or without mitochondrially targeted *Av*NifS and *Av*NifU (SL133, Table 1). Henceforth, Nif/Anf proteins expressed in and purified from *N. benthamiana* will have the designation ‘*Nb-*’ at the front of the proteins. Three days after agroinfiltration, the plants were moved into the dark and left overnight, and leaves harvested the following morning in the dark to prevent oxygen being generated from photosynthesis. SWATH proteomic analysis of the soluble fraction of *N. benthamiana* leaf infiltrated with the three *Nb*-*Ko*NifHHv2 combinations (*Nb*-*Ko*NifHHv2 only, *Nb*-*Ko*NifHHv2_ *Nb*-*Ko*NifM, or *Nb*-*Ko*NifHHv2_ *Nb*-*Ko*NifM_ *Nb*-*Av*NifS_ *Nb*-*Av*NifU) confirmed expression of all Nif proteins (Supplementary Fig. 10). Furthermore, the MPP-cleaved residual tryptic peptide ‘ISTQVVR’ was also detected in all soluble fractions, indicating evidence of MPP processing of *Nb*-*Ko*NifHHv2 (Supplementary Fig. 10). Isolation of *Nb*-*Ko*NifHHv2 under anoxic conditions by affinity chromatography gave on average 1.07 mg/kg fresh leaf, with an average purity of 27% (see Supplementary Fig. 11). The placement of the Twin-Strep-tag® within the linker between the NifH monomers allowed for purification, although some of the target protein was occasionally detected in the flowthrough fractions. Some degradation of purified *Nb*-*Ko*NifHHv2 was consistently observed (Supplementary Fig. 11). This may have been due to the purification process, as degradation was not detected during the initial testing of the solubility of *Nb*-*Ko*NifHHv2 (Supplementary Fig. 5).

**Fig. 2:**
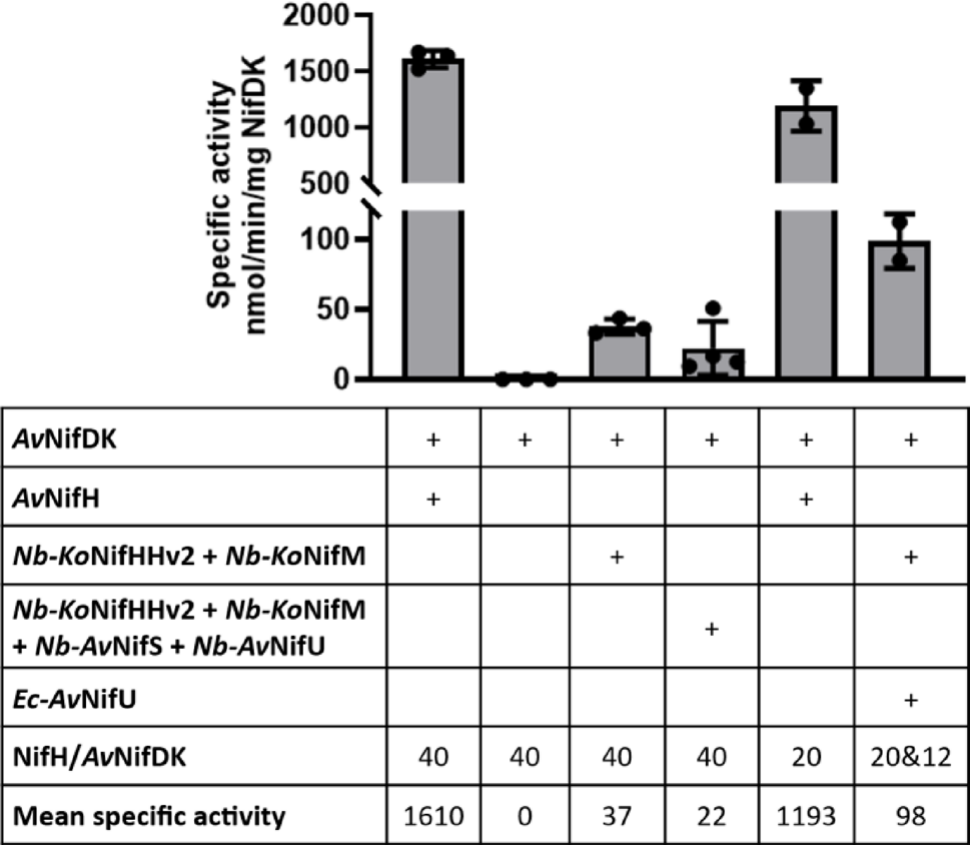
Acetylene reduction assay (ARA) of *Nb*-*Ko*NifHHv2 (SN656) isolated *from N. benthamiana* mitochondria. Specific activity calculated from ethylene production (nmol/min/mg *Av*NifDK) from acetylene of bacterial *Av*NifDK combined with bacterial *Av*NifH or plant-expressed *Nb*-*Ko*NifHHv2, which was coexpressed with *Nb*-*Ko*NifM (SN360) with or without *Nb*-*Av*NifS and *Nb-Av*NifU (SL133). [4Fe-4S] cluster reconstitution of *Nb*-*Ko*NifHHv2 was achieved via *Av*NifU expressed and purified from *Escherichia coli* that was chemically reconstituted with iron-sulfur clusters (*Ec-Av*NifU). Each data point of ethylene production by *Nb*-*Ko*NifHHv2 and *Av*NifDK represents a biological replicate, and those of bacterial *Av*NifH or *Av*NifDK represents technical replicates. The ratio of *Av*/ *Nb*-*Ko*NifH:*Av*NifDK was 40:1 for the as-isolated *Nb*-*Ko*NifHHv2 ARA (first bar is *Av*NifH:*Av*NifDK control = 40:1). For the iron-sulfur cluster-reconstituted ARA the *Av*NifH:*Av*NifDK control was 20:1 (fifth bar), and the technical replicates of *Nb*-*Ko*NifH : *Av*NifDK were 12 and 20:1. Details of constructs are summarised in Table 1

Function of *Nb*-*Ko*NifHHv2 protein isolated from *N. benthamiana* leaf was assessed using the acetylene reduction assay (ARA). *Nb*-*Ko*NifHHv2 was active as-isolated, producing ethylene at 37 nmol/min/mg NifDK (Fig. 2, n = 3), which was approximately 2% of the positive control which had purified *Av*NifH and *Av*NifDK isolated from *A. vinelandii* strain DJ2102 (kindly donated by Professor Dennis Dean). Significantly, co-expression of *Nb*-*Av*NifS and *Nb*-*Av*NifU within the plant mitochondria did not increase the activity of *Nb*-*Ko*NifHHv2 (22 nmol/min/mg *Av*NifDK of ethylene produced, n = 4, Fig. 2). However, when the isolated *Nb*-*Ko*NifHHv2 protein was incubated in vitro with purified *Av*NifU from *E. coli* (*Ec-Av*NifU) that was chemically loaded with iron-sulfur clusters, activity was improved to approximately 8% of the positive control (98 nmol/min/mg *Av*NifDK of ethylene produced, Fig. 2), a ~ fourfold increase from the as-isolated rates. It was concluded that at least some of the isolated *Nb*-*Ko*NifHHv2 protein was inactive as-isolated but re-activatable with *Ec-Av*NifU.

### *Ko*NifHHv2 expressed in *K. oxytoca* nif system in *E. coli* supports diazotrophic growth

**Fig. 3:**
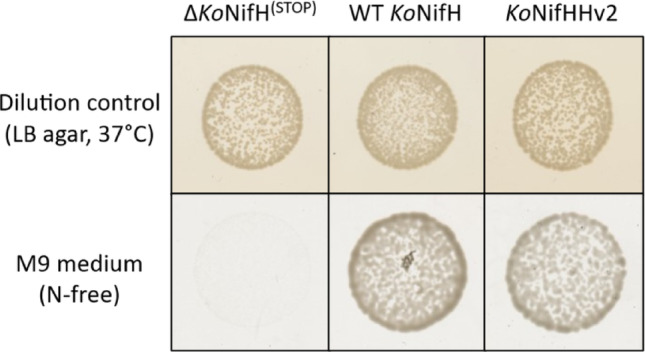
*Ko*NifHHv2 supports diazotrophic growth in *E. coli*. Diazotrophic growth of *E. coli* BL21 Star strains expressing the complete *Klebsiella oxytoca nif* operon on M9 medium lacking fixed nitrogen (N-free). Growth of a *KonifH*-knockout strain (Δ*Ko*NifH^(STOP)^) and a strain expressing the translational fusion *Ko*NifHHv2 were compared to the wildtype (WT) *Ko*NifH. Cells were spotted at equal density onto N-free M9 medium and incubated for seven days under anaerobic conditions. A dilution control was grown aerobically on LB agar at 37 °C overnight to compare initial cell density of the three *E. coli* strains

To determine whether *Ko*NifHHv2 retains the canonical functions of NifH required for nitrogenase activity, including electron transfer to dinitrogenase and maturation of the metalloclusters required for an active NifDK complex, we tested whether *Ko*NifHHv2 in the *K. oxytoca nif* system could support diazotrophic growth in *E. coli* (Fig. 3). To do this, we generated a Golden Gate-compatible variant of the pKU7017 plasmid containing the full *Klebsiella oxytoca nif* operon (*nifJHDKENTYWUSVXZMFBQLA*), with a GFP dropout cassette inserted in place of *nifH*. The dropout cassette was replaced with either the wildtype (WT) *Ko*NifH coding sequence, the *Ko*NifHHv2 coding sequence, or a full-length *Ko*NifH coding sequence containing two stop codons near the 5’ end. *E. coli* with the *K. oxytoca nif* system containing WT*KonifH* supported anaerobic growth on nitrogen-free M9 medium. Disruption of WT*KonifH* by introduction of stop codons abolished growth under the same conditions, confirming loss of nitrogenase activity. Replacement of *KonifH* with *KonifHHv2* restored diazotrophic growth to levels comparable to WT*KonifH* under nitrogen-free conditions. This indicates that *Ko*NifHHv2 is functionally competent in vivo and supports operation of the nitrogenase pathway, consistent with retention of the canonical functions of NifH required for nitrogen fixation-dependent growth.

### A translational fusion of two *Av*AnfH monomers is also soluble in plant mitochondria

**Fig. 4:**
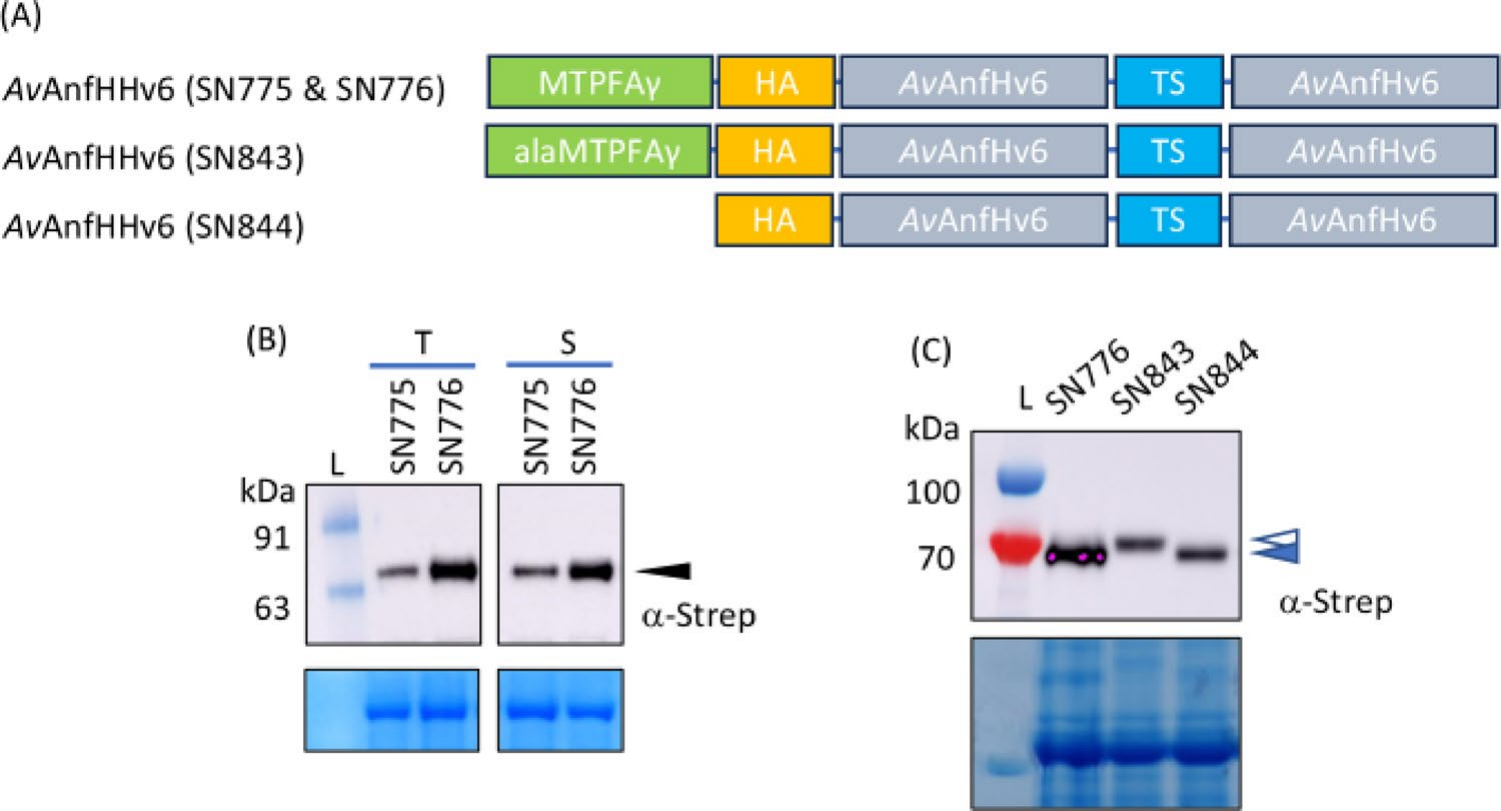
Assessing solubility and processing of translationally fused variant *Av*AnfH dimers (*Av*AnfHHv6) targeted to *N. benthamiana* mitochondria. **A** Configuration of *Av*AnfHHv6 translational fusions used for this analysis (MTPFAγ, first 51 amino acid residues of *Arabidopsis thaliana* γATPase preprotein; HA, hemagglutinin tag; TS, Twin-Strep-tag®; genetic parts in diagram are not to scale); **B** Anti-strep western blot (top) and corresponding Coomassie-stained SDS-PAGE (bottom) of total (T), soluble (S), and insoluble (I) fractions of *Nb*-*Av*AnfHHv6 expressed from SN775 (with the 35S terminator at the C terminus of *AvanfHHv6*) or SN776 (with the TTm transcription terminator at the C terminus of *AvanfHHv6*), black triangle denotes *Nb*-*Av*AnfHHv6; **C** Anti-strep western blot (top) and corresponding Coomassie-stained SDS PAGE (bottom) to show mitochondrial targeting peptide processing of *Nb*-*Av*AnfHHv6 by the mitochondrial processing peptidase (MPP). Total fractions were used for this analysis. Open triangle denotes signals corresponding to unprocessed *Av*AnfHHv6 (SN843), and solid blue triangle denotes signals corresponding to the size of MPP-processed *Nb*-*Av*AnfHHv6 (SN776). L, protein ladder. Full-length blot and gel images are shown in Supplementary Fig. 13A for (B), and Supplementary Fig. 13B for (C). Details of constructs are summarised in Table 1

We have previously shown that *Av*AnfH was only partially soluble when targeted to plant mitochondria (Johnston et al. 2023). Based on the increased solubility of the *Nb-Ko*NifHH and *Nb-Av*NifHH translational fusion dimers targeted to *N. benthamiana* mitochondria, the dimerization study was extended to the wildtype dinitrogenase reductase from the iron-only nitrogenase, AnfH, from *A. vinelandii* (*Av*AnfHwt). In an initial dimerization experiment, an expression construct coding for a translationally fused dimer of *Av*AnfH (*Av*AnfHHwt) was generated having an analogous structure to *Ko*NifHHv1, including MTPFAγ, HA epitope, and the intervening ArsA linker. When expressed in *N. benthamiana* leaf from a construct having the e35S promoter and TTm transcription terminator, *Nb-Av*AnfHHwt was produced only in partially soluble form when targeted to the mitochondria (Supplementary Fig. 12). A recent study from our lab showed that a synthetic *Av*AnfH monomer variant (*Av*AnfHv6) with three amino acid substitutions from the wildtype protein sequence was fully soluble but not active as-isolated from *N. benthamiana* leaf (Gregg et al. 2025a). We generated a translational fusion of two *Av*AnfHv6 monomers to see if it could assist in loading of [4Fe-4S] clusters within plant mitochondria, since the *Nb*-*Ko*NifHHv2 protein was both soluble and partially loaded with [4Fe-4S] clusters based on its activity. This translational fusion had two *Av*AnfHv6 monomers linked by a Twin-Strep-tag®, and MTPFAγ and HA epitope added at the *N*-terminus, resulting in a final configuration of MTPFAγ::HA::*Av*AnfHv6::TS::*Av*AnfHv6 (*Av*AnfHHv6, Fig. 4A, Table 1).

Two expression constructs were made for the *Av*AnfHHv6 proteins – one with the e35S promoter and 35S transcription terminator (SN775), and the other with the e35S promoter and the TTm transcription terminator (SN776). Both constructs were agroinfiltrated into *N. benthamiana* leaf in the absence of *Av*NifS and *Av*NifU and resulting protein expression analysed by western blot. This showed that both constructs expressed *Nb*-*Av*AnfHHv6 in completely soluble form when targeted to plant mitochondria, with greater expression from the construct containing the TTm transcription terminator (Fig. 4B, Supplementary Fig. 13A). Mitochondrial targeting of the dimeric *Nb*-*Av*AnfHHv6 protein was also investigated in the same manner as for *Nb*-*Ko*NifHHv1&2 by designing two molecular weight control constructs for *Nb*-*Av*AnfHHv6 – one that had the alanine substituted MTPFAγ variant to prevent mitochondrial processing by MPP (alaMTPFAγ::*Av*AnfHHv6; SN843) and the other that produced a cytosolic protein about the size of the MPP-processed form of *Av*AnfHH (HA::*Av*AnfHHv6, SN844). Anti-strep western blot analysis of the total fraction of *N. benthamiana* leaves expressing *Nb*-MTP*Av*AnfHHv6 predominantly showed a faster migrating signal in line with the cytosolically targeted version (Fig. 4C, Supplementary Fig. 13B), indicating successful targeting and processing by MPP of the protein within the leaf mitochondrial matrix.

### *Nb-Av*AnfHHv6 is not active as-isolated when purified from plant mitochondria

**Fig. 5:**
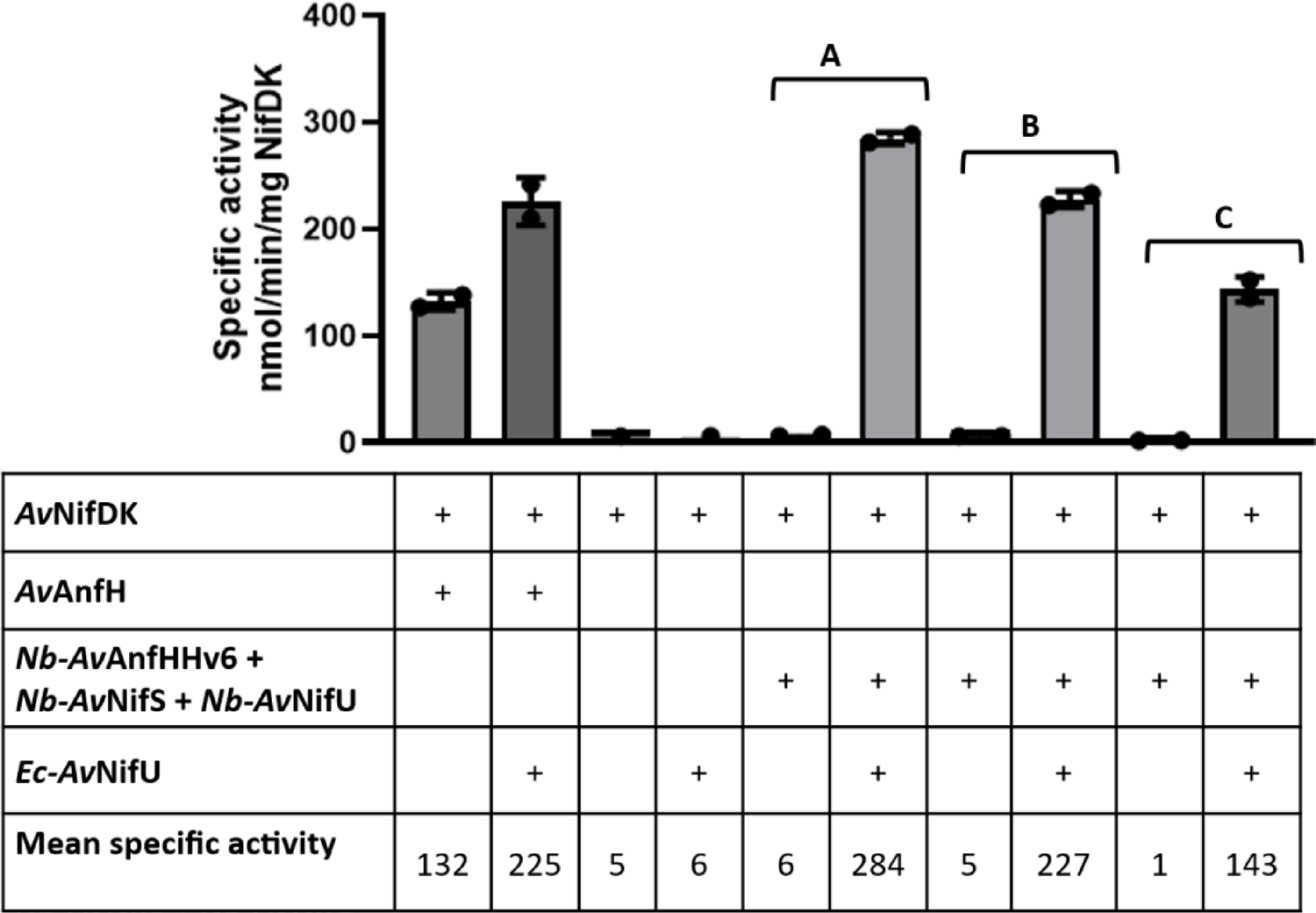
Acetylene reduction assay of *Nb*-*Av*AnfHHv6 (SN776) isolated from *N. benthamiana* mitochondria. Specific activity was calculated from ethylene production (nmol/min/mg *Av*NifDK) from acetylene of bacterial *Av*NifDK combined with bacterial *Av*AnfH or plant-expressed *Nb*-*Av*AnfHHv6, which was coexpressed with *Nb*-*Av*NifS and *Nb-Av*NifU (SL133). In vitro [4Fe-4S] cluster reconstitution of *Nb*-*Av*AnfHHv6 was achieved via *Av*NifU expressed and purified from *E. coli* that was chemically reconstituted with iron-sulfur clusters (*Ec-Av*NifU). Each bar of specific activity by *Nb*-*Av*AnfHHV6 combined with *Av*NifDK represents a biological replicate (**A**, **B**, **C**), and all data points on each bar represent technical replicates. The ratio of *Av*AnfH:*Av*NifDK was 20:1. Details of constructs are summarised in Table 1

The activity of *Nb*-*Av*AnfHHv6 protein purified from *N. benthamiana* leaf was assessed by ARA. Purification under anaerobic conditions of the *Nb*-*Av*AnfHHv6 protein from *N. benthamiana* leaves that was co-expressed with *Nb*-*Av*NifS and *Nb*-*Av*NifU (SL133) typically resulted in 10 mg of *Nb*-*Av*AnfHHv6/kg fresh leaf, with approximately 70% of the isolated protein being the dimeric *Nb*-*Av*AnfHHv6 (Supplementary Fig. 14). *Nb*-*Av*AnfHHv6 yields were therefore greater than *Nb*-*Ko*NifHHv2, being typically ~ 10 × more abundant and ~ 3 × times higher purity, with less target protein detected in the flowthrough fractions. As seen with purified *Nb*-*Ko*NifHHv2, there was evidence of degradation of *Nb*-*Av*AnfHHv6 after concentrating the protein. We also attribute this to the purification process, as initial solubility testing has not shown evidence of degradation of *Nb*-*Av*AnfHHv6 (Supplementary Fig. 14). Despite the higher abundance and purity of *Nb*-*Av*AnfHHv6, it did not produce ethylene as-isolated in the in vitro ARA when combined with *Av*NifDK isolated from *A. vinelandii* (Fig. 5). After treatment in vitro with iron-sulfur cluster-loaded *Ec-Av*NifU, however, *Nb*-*Av*AnfHHv6 produced as much ethylene (specific activity 218 nmol/min/mg NifDK) as the bacterial *Av*AnfH/*Av*NifDK control (specific activity 225 nmol/min/mg NifDK), indicating that the plant-produced *Nb*-*Av*AnfHHv6 protein could be efficiently reactivated with [4Fe-4S] clusters from *Ec-Av*NifU.

## Discussion

Despite the abundance of nitrogenase research from *A. vinelandii* and *K. oxytoca*, many of these well-characterised proteins have proven difficult to express in eukaryotes in a soluble and functional form when targeted to the mitochondria (Allen et al. 2020; Buren et al. 2017, 2019; Johnston et al. 2023; Okada et al. 2020). In this study we have successfully demonstrated the use of a translational fusion of two NifH monomers to produce a soluble dinitrogenase reductase of the MoFe system when targeted to this organelle. This was demonstrated for translational fusion dimers of both *K. oxytoca* and *A. vinelandii* NifH, *Ko*NifHH and *Av*NifHH. Solubility of these proteins substantially increased when NifM was co-expressed in the plant cells. Purified *Nb*-*Ko*NifHH demonstrated partial activity as-isolated, indicating some degree of iron-sulfur cluster loading in plant mitochondria at ambient oxygen conditions. Significantly, the *Ko*NifHH activity was not dependent on co-expression of the nitrogenase-specific iron-sulfur cluster machinery NifS and NifU, indicating that plant mitochondria are endogenously capable of some iron-sulfur cluster loading onto NifH at ambient oxygen concentrations. The activity was further increased by in vitro treatment of the purified protein with *Ec-Av*NifU that was chemically loaded with iron-sulfur clusters. In contrast, the translational fusion of two dinitrogenase reductase variant monomers of the *A. vinelandii* FeFe system, *Nb-Av*AnfHHv6, was not active as-isolated from plant mitochondria but was able to be activated when reconstituted with iron-sulfur clusters in vitro.

In this study *Ko*NifH only became mostly soluble in plant mitochondria when two NifH monomers were translationally fused. Dinitrogenase reductase, including NifH and AnfH, function as homodimers with the [4Fe-4S] cluster bridging the two monomers (Georgiadis et al. 1992; Trncik et al. 2022). In addition to the [4Fe-4S] cluster, there are amino acid residues of the opposing *Av*NifH monomers along the dimer interface that support hydrogen bonds and salt-bridge interactions (Schlessman et al. 1998). In natural hosts such as *K. oxytoca*, NifH is expressed at a high level during diazotrophy, sometimes accumulating to as much as 10% of the total protein (Waite et al. 2021). This high concentration of NifH in their natural host may help monomers to locate partners and form homodimers, in addition to sufficient supply of [4Fe-4S] clusters that could be incorporated to bridge the two NifH monomers and stabilise the homodimer structure. Our results suggest that a translational fusion of NifH may assist in homodimer formation due to the physical proximity of two monomers when the protein abundance is relatively low. Once in the homodimer configuration, solubility of NifH is improved and therefore stability of the protein may also be improved within the plant mitochondria, allowing [4Fe-4S] clusters to be inserted. Interestingly the translational fusion of two wildtype *Av*AnfH monomers did not improve solubility when targeted to this organelle. This may be due to wildtype *Av*AnfH being more prone to misfolding and/or aggregating, or lack of a chaperone protein such as NifM, the requirement of which could be advantageous for proper folding and dimerization of NifHs when expressed in a heterologous environment.

Along with previous reports, our study has shown that *Nb-*NifM was functional and required for maturation of *Nb*-NifHH translational fusions, by evidence of the majority of *Ko*- and *Av*NifHH becoming soluble only when NifM was co-expressed in plant mitochondria. This observation is analogous to the requirement of *Av*NifM for producing a soluble *Av*NifH that was targeted to tobacco chloroplasts (Eseverri et al. 2020). Based on amino acid sequence similarity, NifM is predicted to be a peptidyl-prolyl cis–trans isomerase that is involved in the maturation of certain NifH proteins, possibly by assisting in protein folding (Gavini et al. 2006). More recently, Solomon et al*.* (2023) conducted a structural analysis of *Av*NifM by comparing its Alphafold structural prediction to other proteins in the same database. They found that the N- and C-termini of the predicted structure of *Av*NifM resembled foldases found in bacteria and suggested that NifM may also function as a chaperone for NifH. The requirement of NifM for NifH abundance, solubility and/or function of MoFe nitrogenase has also been reported for *K. oxytoca*, *A. vinelandii, Geobacter sulfurreducens,* and *Rhizobium meliloti* NifH (Eseverri et al. 2020; Howard et al. 1986; Jacobson et al. 1989; Petrova et al. 2002; Yang et al. 2014). Further, Ito et al. (2024) reported increased NifH solubility and nitrogenase activity by over-expressing NifM in *E. coli* along with 17 other Nif and related proteins, confirming a role for NifM in correct folding of NifH for activity. It is noteworthy here that the isomeric modification that NifM provides to NifHH in the plant cytosol can be maintained after transport of the protein into the mitochondrial matrix. Reversion of isomerisation reactions are relatively slow, estimated at 2.5 × 10^–3^ s^−1^ (Grathwohl and Wuthrich 1981). Therefore, if NifM functions as an isomerase, the modification it makes to the NifHH translational dimer may persist until entry into the mitochondrial matrix and refolding to a soluble conformation. However, preliminary experiments have not been able to obtain activity as-isolated of *Nb*-*Ko*NifHH co-expressed with cytosolic *Nb*-*Ko*NifM (data not shown). This result indicates that there may be additional functions of NifM that require it to be present when NifH is being refolded in the mitochondria.

Most importantly we have shown that *Ko*NifHH targeted to plant mitochondria can be isolated in a functional form, joining a short list of reports having isolated an active NifH from plants. *Hydrogenobacter thermophilus* NifH isolated from *N. benthamiana* leaf mitochondria produced low levels of ethylene (12–28 nmol ethylene/min/mg *Av*NifDK) in an ARA (Jiang et al. 2021). Similarly, *H. thermophilus* NifH isolated from stably transformed rice leaf and callus mitochondria produced approximately 7 and 60 nmol ethylene/min/mg *Av*NifDK, respectively (Baysal et al. 2022). In contrast*, A. vinelandii* NifH that was purified from *N. benthamiana* chloroplasts generated up to 189 nmol ethylene/min/mg *Av*NifDK (Eseverri et al. 2020; Ivleva et al. 2016). While there may be differences in the compatibility between the various plant-isolated NifHs and *Av*NifDK used in those studies, as well as differences in the assay buffers, incubation conditions and analysis methods used, it is worth noting that the purified *Nb-Ko*NifHH here was about as active (37 nmol ethylene/min/mg *Av*NifDK) as the *H. thermophilus* NifH expressed and isolated from *N. benthamiana* and rice mitochondria. In contrast to plant-expressed NifH, those expressed in yeast mitochondria yielded considerably higher activity, producing 400–2000 nmol ethylene/min/mg *Av*NifDK (Jiang et al. 2021; Lopez-Torrejon et al. 2021, 2016). This difference in activity levels of NifH or AnfH between plants and yeast may be due to differences in iron availability and oxygen levels of the microenvironment where dinitrogenase reductase is expressed. The two yeast studies used culture medium supplemented with iron, and dissolved oxygen levels were near zero at the time of induction of NifH expression, despite them being grown in aerobic conditions (Lopez-Torrejon et al. 2021, 2016). More available iron to the yeast cells may increase [4Fe-4S] cluster loading onto NifH and AnfH, and the microaerobic environment would better protect [4Fe-4S] cluster-loaded NifH from oxidative damage, which may have contributed to the higher activity of dinitrogenase reductase purified from yeast mitochondria.

One possible reason for some of the *Ko*NifHH being in functional form in plant mitochondria is that the close association of the two *Ko*NifH monomers assists with the insertion of [4Fe-4S] clusters. We tested this hypothesis with a soluble *Av*AnfH variant, *Av*AnfHv6, that was not functional as-isolated from plant mitochondria when expressed as a monomer (Gregg et al. 2025a). Despite our hopes that the translational fusion of two *Av*AnfHv6 monomers could become functional in plant mitochondria, we did not see activity of this protein as-isolated. *Av*AnfH is reported to be more sensitive to oxygen compared to NifH (Chisnell et al. 1988; Moshiri et al. 1994), which may be a factor contributing to *Nb-Av*AnfHHv6 being non-functional upon isolation from leaf tissue, which is an oxygen-rich environment. It may be interesting to investigate the activity of *Av*AnfHHv6 in physiologically hypoxic plant tissue such as shoot and root apical meristems (Weits et al. 2021). Coexpression of additional components to scrub oxygen such as Anf3 may also be needed to further decrease oxygen levels in the microenvironment where nitrogenase is expressed (Varghese et al. 2019). However, it is promising that *Nb-Av*AnfHHv6 can be made functional when reconstituted with iron-sulfur clusters in vitro, indicating that the protein is folded in its correct form and able to receive iron-sulfur clusters when available.

Our study found that the activity of isolated *Ko*NifHH did not increase by co-expressing NifS and NifU in plant mitochondria. Contrary to the requirement for NifS and NifU for a functional NifH in *A. vinelandii* (Jacobson et al. 1989), and evidence of NifU transferring iron-sulfur clusters to NifH in vitro (Dos Santos et al. 2004), NifU was not essential for obtaining functional NifH expressed in several recombinant systems. For example, it has been shown that NifS and NifU are not needed for loading iron-sulfur clusters onto NifH expressed in *E. coli* (Howard et al. 1986; Solomon et al. 2023), or in the mitochondria of yeast or plants (Baysal et al. 2022; Lopez-Torrejon et al. 2021, 2016). Furthermore, Dos Santos et al. (2007) has shown diazotrophic growth in *A. vinelandii* when NifS and NifU were replaced with high levels of expression of housekeeping iron sulfur cluster (ISC) components IscS and IscU. This highlights the ability of the ISC machinery in loading iron-sulfur clusters onto NifH, and that the mitochondrial ISC system is the likely source of iron-sulfur clusters that were loaded onto *Nb-Ko*NifHH.

Along with others, this study points to a paradox in mitochondrial expression of NifH homologues that NifS and NifU do not seem to be loading iron-sulfur clusters onto them, yet these NifH homologues isolated from plants can be rapidly loaded with iron-sulfur clusters donated from NifU in vitro. Some possible reasons for this paradox include (i) lack of sufficient iron provision to NifU, rendering it non-active in plant mitochondria, or (ii) iron-sulfur clusters formed on NifU are indiscriminately transferred to other endogenous metalloproteins, such as aconitase in the mitochondria, (iii) mitochondria is not anaerobic enough for NifU to function (Rapson et al. 2026), or (iv) NifH is not able to receive iron-sulfur clusters from NifU in plant mitochondria, and may require additional components such as the Rnf1 complex (Curatti et al. 2005) or more ATP (Chen et al. 1994). Past attempts to isolate a fully as-isolated NifU from bacteria and plants have been proven difficult (Jiang et al. 2021; Dos Santos et al. 2004; Agar et al. 2000; Yuvaniyama et al. 2000). It is hypothesised that the transient iron-sulfur clusters on NifU, if present in the expression host, are lost upon purification. Expression and purification of a mutant NifU that has a modified iron-sulfur cluster binding site that prevents release of the transient iron-sulfur clusters could be informative to confirm loading of the transient iron-sulfur clusters onto NifU. A functional iron-sulfur cluster assembly and transfer pathway, whether it be NifS and NifU proteins, or increased expression of the endogenous ISC system in mitochondria, or any other pathway that is capable of loading iron-sulfur clusters onto nitrogenase components are a prerequisite for nitrogenase function. Further work is needed to overcome this bottleneck of iron-sulfur cluster provision to obtain a fully active NifH in plant mitochondria.

## Supporting information

The following supporting information are provided: Supporting_Information.pdf contains Supplementary Figs. 1–14. NbKoNifHH_protein_matrix.xlsx contains proteomic data for *N. benthamiana* expression of *Ko*NifHHv2, *Ko*NifM, *Av*NifS and *Av*NifU.

## Supplementary Information

Below is the link to the electronic supplementary material.

Supplementary Material 1

Supplementary Material 2

## Data Availability

The data supporting the conclusions of this article are available in the Supporting information of this article.
