# Supplementary material for: Use of translational fusions to express functional Klebsiella oxytoca dinitrogenase reductase in plant mitochondria
Source: Plant Mol Biol. 2026 Jul 20;116(4):71. doi: 10.1007/s11103-026-01735-5 (PMC13385145; doi:10.1007/s11103-026-01735-5)
Supplement: Supplementary file 1 — Supplementary Material 1 [file 11103_2026_1735_MOESM1_ESM.pptx]

## Slide 1
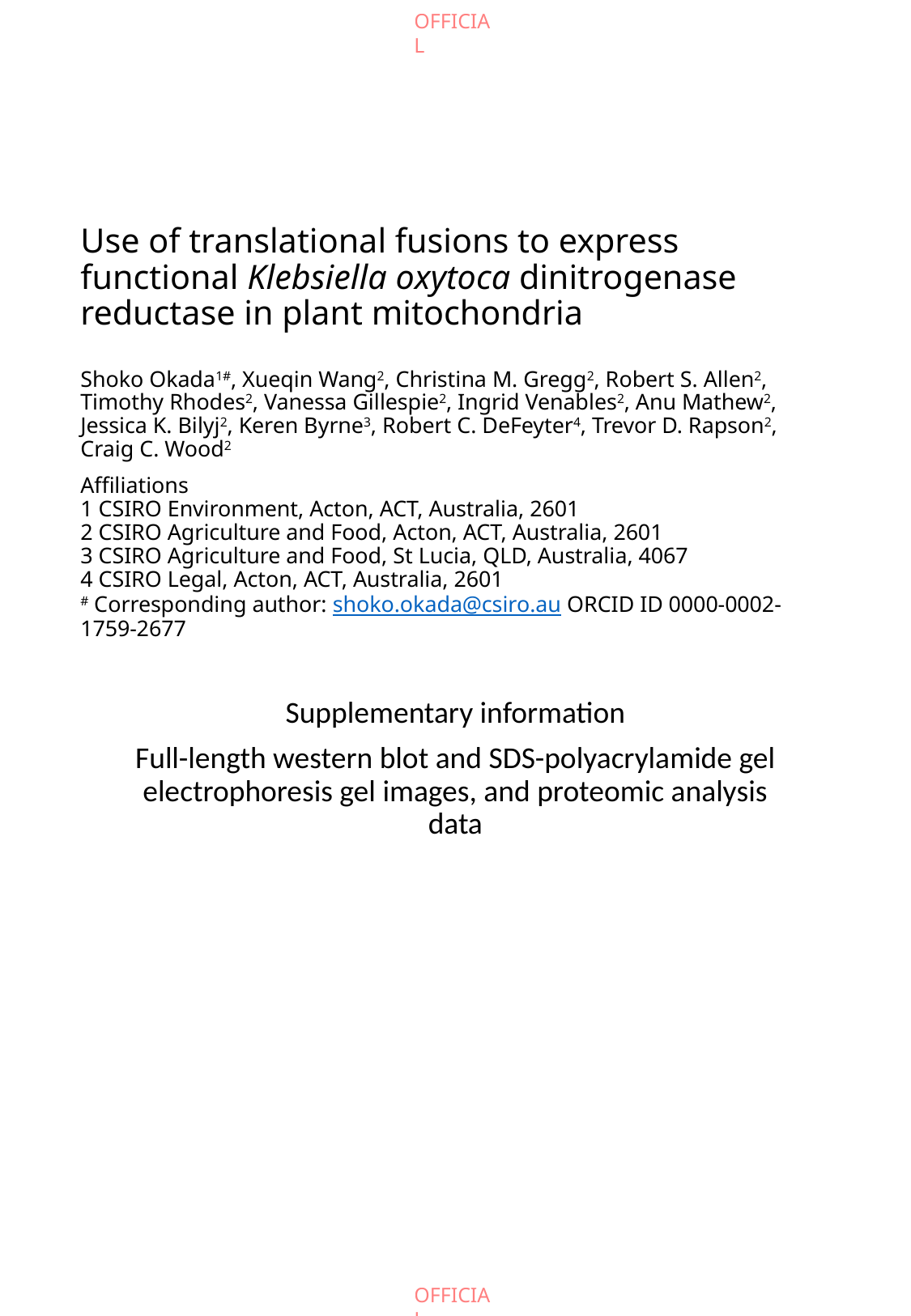

# Use of translational fusions to express functional Klebsiella oxytoca dinitrogenase reductase in plant mitochondria Shoko Okada1#, Xueqin Wang2, Christina M. Gregg2, Robert S. Allen2, Timothy Rhodes2, Vanessa Gillespie2, Ingrid Venables2, Anu Mathew2, Jessica K. Bilyj2, Keren Byrne3, Robert C. DeFeyter4, Trevor D. Rapson2, Craig C. Wood2 Affiliations 1 CSIRO Environment, Acton, ACT, Australia, 2601 2 CSIRO Agriculture and Food, Acton, ACT, Australia, 2601 3 CSIRO Agriculture and Food, St Lucia, QLD, Australia, 4067 4 CSIRO Legal, Acton, ACT, Australia, 2601 # Corresponding author: shoko.okada@csiro.au ORCID ID 0000-0002-1759-2677
Supplementary information
Full-length western blot and SDS-polyacrylamide gel electrophoresis gel images, and proteomic analysis data

## Slide 2
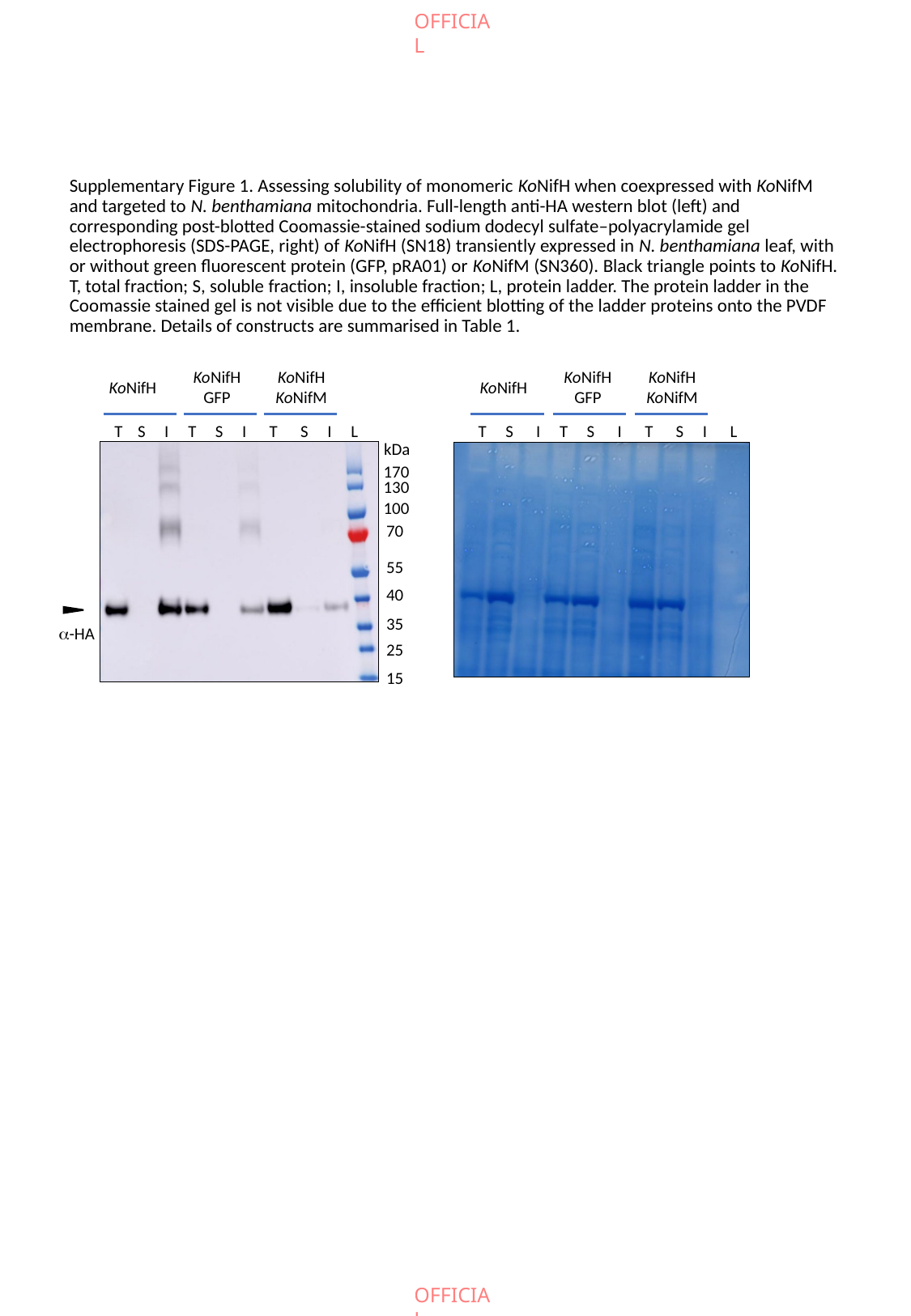

Supplementary Figure 1. Assessing solubility of monomeric KoNifH when coexpressed with KoNifM and targeted to N. benthamiana mitochondria. Full-length anti-HA western blot (left) and corresponding post-blotted Coomassie-stained sodium dodecyl sulfate–polyacrylamide gel electrophoresis (SDS-PAGE, right) of KoNifH (SN18) transiently expressed in N. benthamiana leaf, with or without green fluorescent protein (GFP, pRA01) or KoNifM (SN360). Black triangle points to KoNifH. T, total fraction; S, soluble fraction; I, insoluble fraction; L, protein ladder. The protein ladder in the Coomassie stained gel is not visible due to the efficient blotting of the ladder proteins onto the PVDF membrane. Details of constructs are summarised in Table 1.
KoNifH
GFP
KoNifH
KoNifM
KoNifH
T S I T S I T S I L
kDa
170
130
100
70
55
40
35
25
15
α-HA
KoNifH
GFP
KoNifH
KoNifM
KoNifH
T S I T S I T S I L

## Slide 3
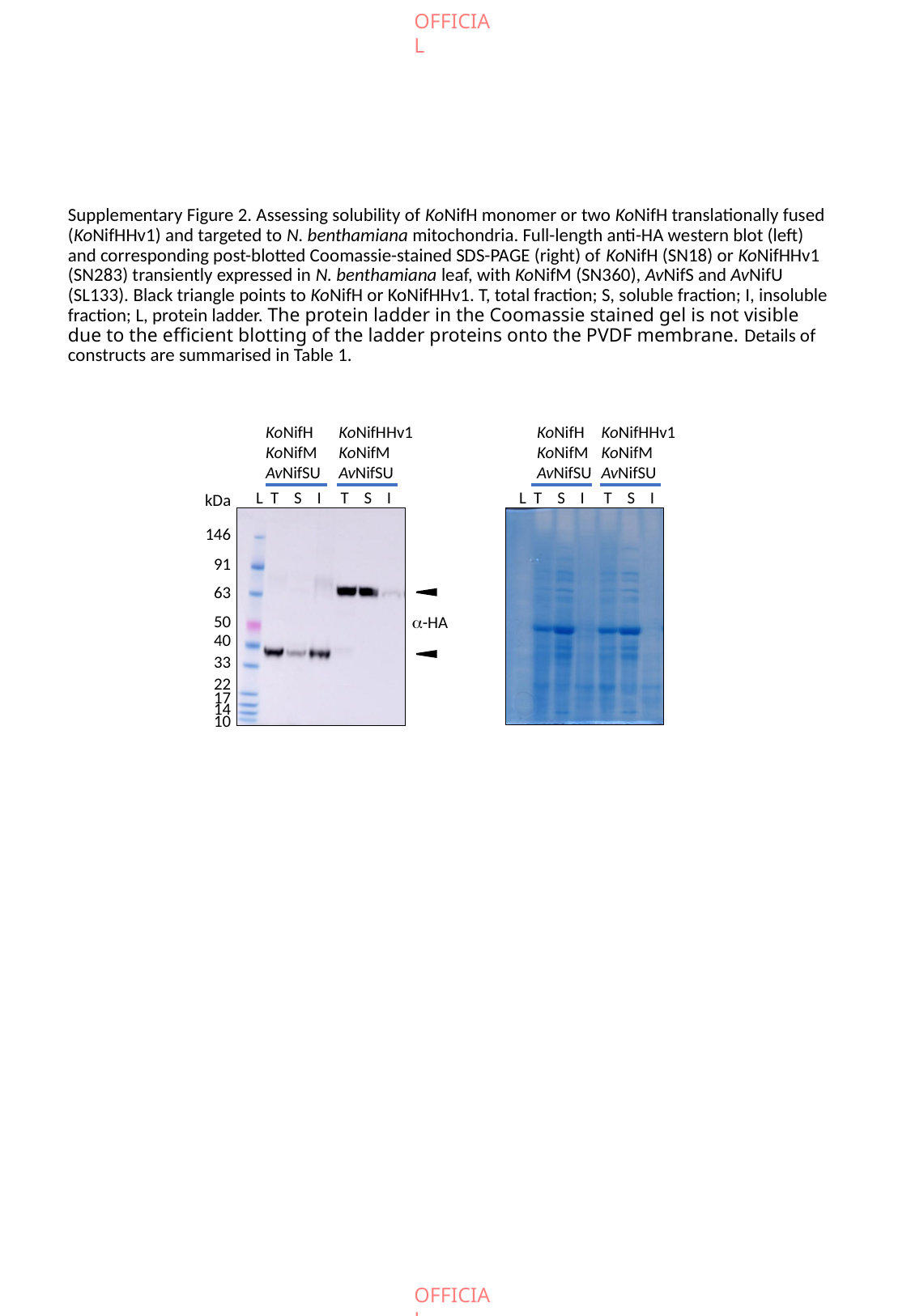

# Supplementary Figure 2. Assessing solubility of KoNifH monomer or two KoNifH translationally fused (KoNifHHv1) and targeted to N. benthamiana mitochondria. Full-length anti-HA western blot (left) and corresponding post-blotted Coomassie-stained SDS-PAGE (right) of KoNifH (SN18) or KoNifHHv1 (SN283) transiently expressed in N. benthamiana leaf, with KoNifM (SN360), AvNifS and AvNifU (SL133). Black triangle points to KoNifH or KoNifHHv1. T, total fraction; S, soluble fraction; I, insoluble fraction; L, protein ladder. The protein ladder in the Coomassie stained gel is not visible due to the efficient blotting of the ladder proteins onto the PVDF membrane. Details of constructs are summarised in Table 1.
KoNifH
KoNifM
AvNifSU
KoNifHHv1
KoNifM
AvNifSU
L T S I T S I
kDa
146
91
63
50
40
33
22
17
14
10
α-HA
KoNifH
KoNifM
AvNifSU
KoNifHHv1
KoNifM
AvNifSU
L T S I T S I

## Slide 4
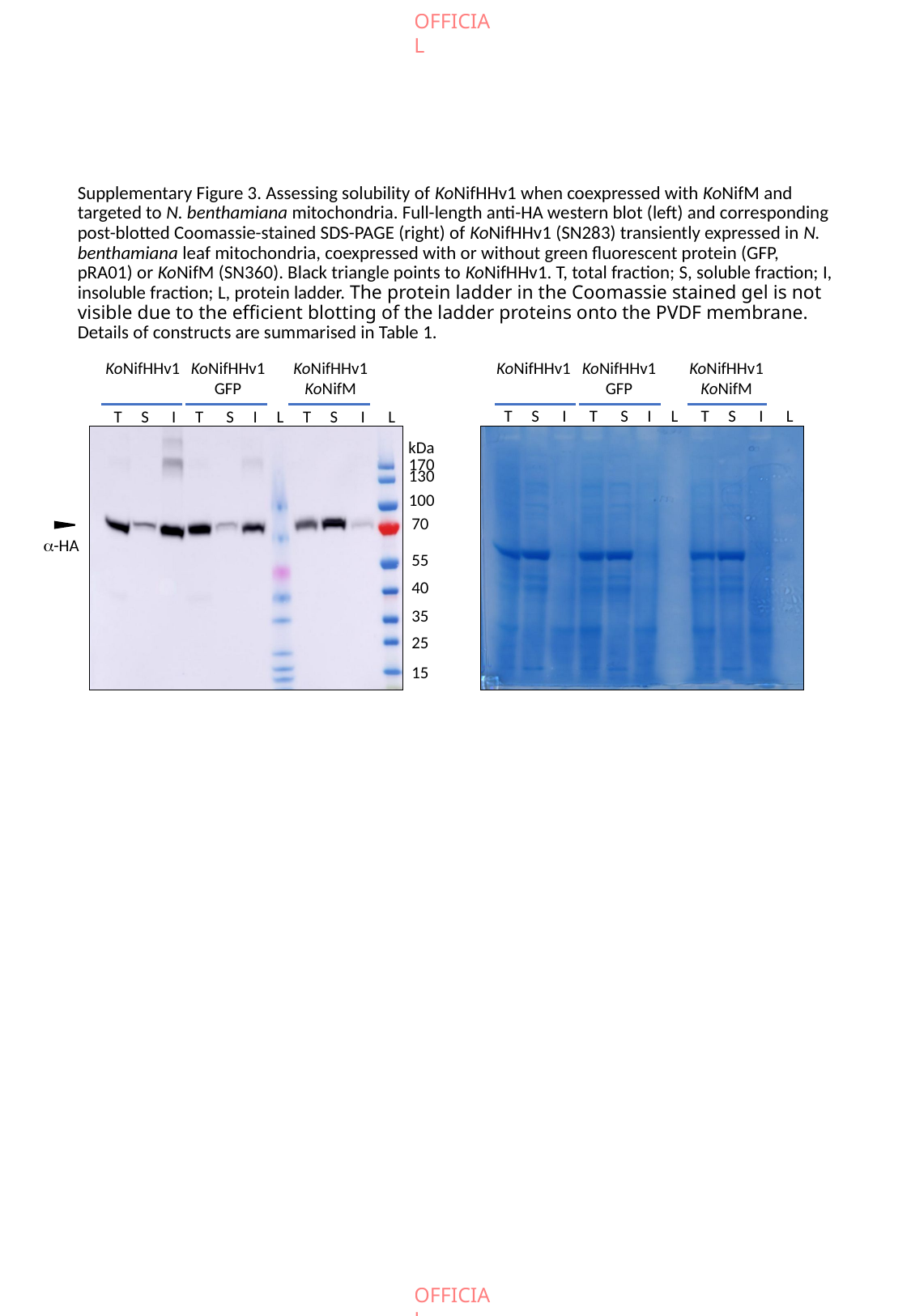

# Supplementary Figure 3. Assessing solubility of KoNifHHv1 when coexpressed with KoNifM and targeted to N. benthamiana mitochondria. Full-length anti-HA western blot (left) and corresponding post-blotted Coomassie-stained SDS-PAGE (right) of KoNifHHv1 (SN283) transiently expressed in N. benthamiana leaf mitochondria, coexpressed with or without green fluorescent protein (GFP, pRA01) or KoNifM (SN360). Black triangle points to KoNifHHv1. T, total fraction; S, soluble fraction; I, insoluble fraction; L, protein ladder. The protein ladder in the Coomassie stained gel is not visible due to the efficient blotting of the ladder proteins onto the PVDF membrane. Details of constructs are summarised in Table 1.
KoNifHHv1
KoNifHHv1
GFP
KoNifHHv1
KoNifM
T S I T S I L T S I L
kDa
170
130
100
70
55
40
35
25
15
α-HA
KoNifHHv1
KoNifHHv1
GFP
KoNifHHv1
KoNifM
T S I T S I L T S I L

## Slide 5
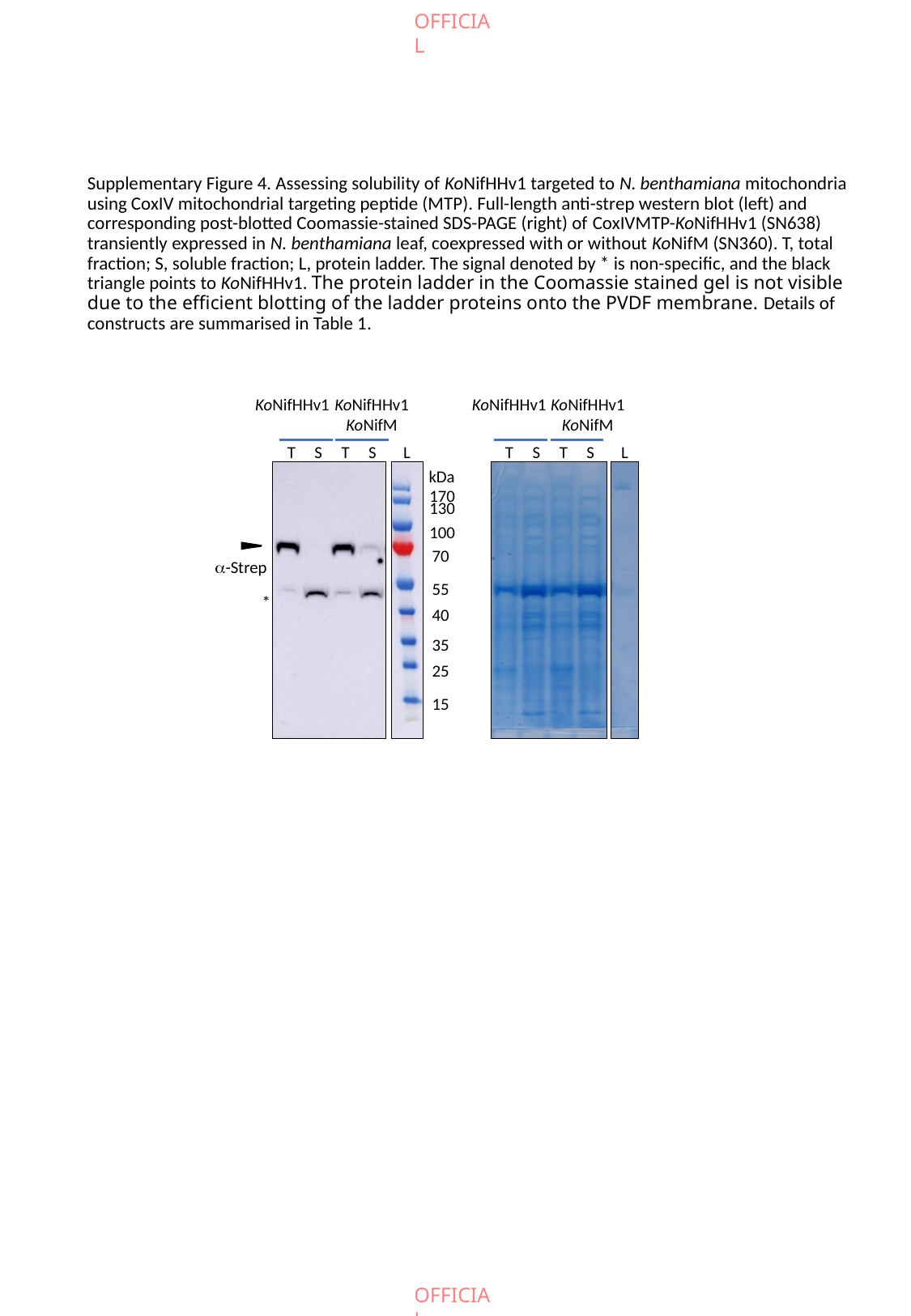

# Supplementary Figure 4. Assessing solubility of KoNifHHv1 targeted to N. benthamiana mitochondria using CoxIV mitochondrial targeting peptide (MTP). Full-length anti-strep western blot (left) and corresponding post-blotted Coomassie-stained SDS-PAGE (right) of CoxIVMTP-KoNifHHv1 (SN638) transiently expressed in N. benthamiana leaf, coexpressed with or without KoNifM (SN360). T, total fraction; S, soluble fraction; L, protein ladder. The signal denoted by * is non-specific, and the black triangle points to KoNifHHv1. The protein ladder in the Coomassie stained gel is not visible due to the efficient blotting of the ladder proteins onto the PVDF membrane. Details of constructs are summarised in Table 1.
KoNifHHv1
KoNifHHv1
KoNifM
T S T S L
kDa
170
130
100
70
55
40
35
25
15
α-Strep
*
KoNifHHv1
KoNifHHv1
KoNifM
T S T S L

## Slide 6
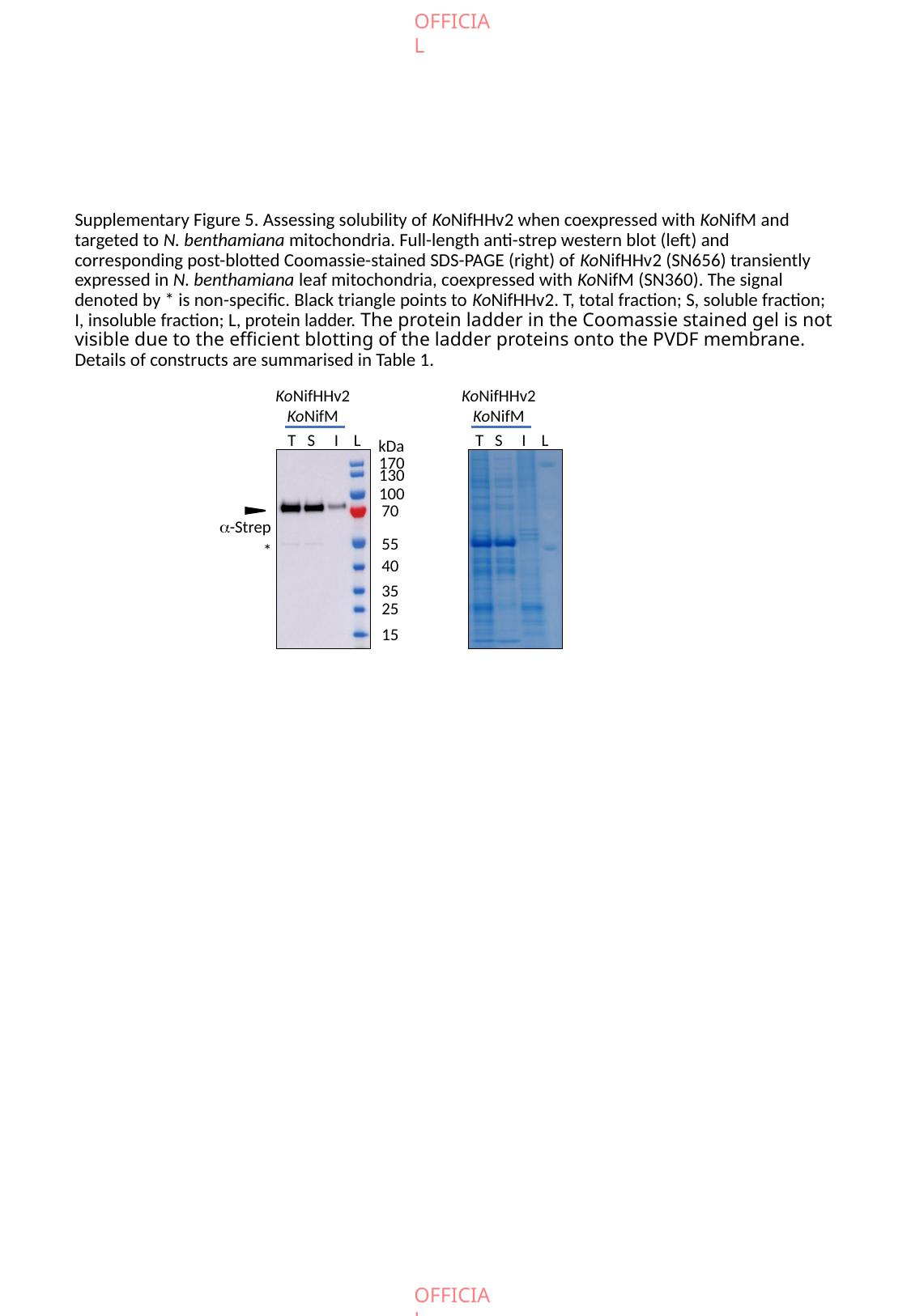

# Supplementary Figure 5. Assessing solubility of KoNifHHv2 when coexpressed with KoNifM and targeted to N. benthamiana mitochondria. Full-length anti-strep western blot (left) and corresponding post-blotted Coomassie-stained SDS-PAGE (right) of KoNifHHv2 (SN656) transiently expressed in N. benthamiana leaf mitochondria, coexpressed with KoNifM (SN360). The signal denoted by * is non-specific. Black triangle points to KoNifHHv2. T, total fraction; S, soluble fraction; I, insoluble fraction; L, protein ladder. The protein ladder in the Coomassie stained gel is not visible due to the efficient blotting of the ladder proteins onto the PVDF membrane. Details of constructs are summarised in Table 1.
KoNifHHv2
KoNifM
T S I L
kDa
170
130
100
70
55
40
35
25
15
α-Strep
KoNifHHv2
KoNifM
T S I L
*

## Slide 7
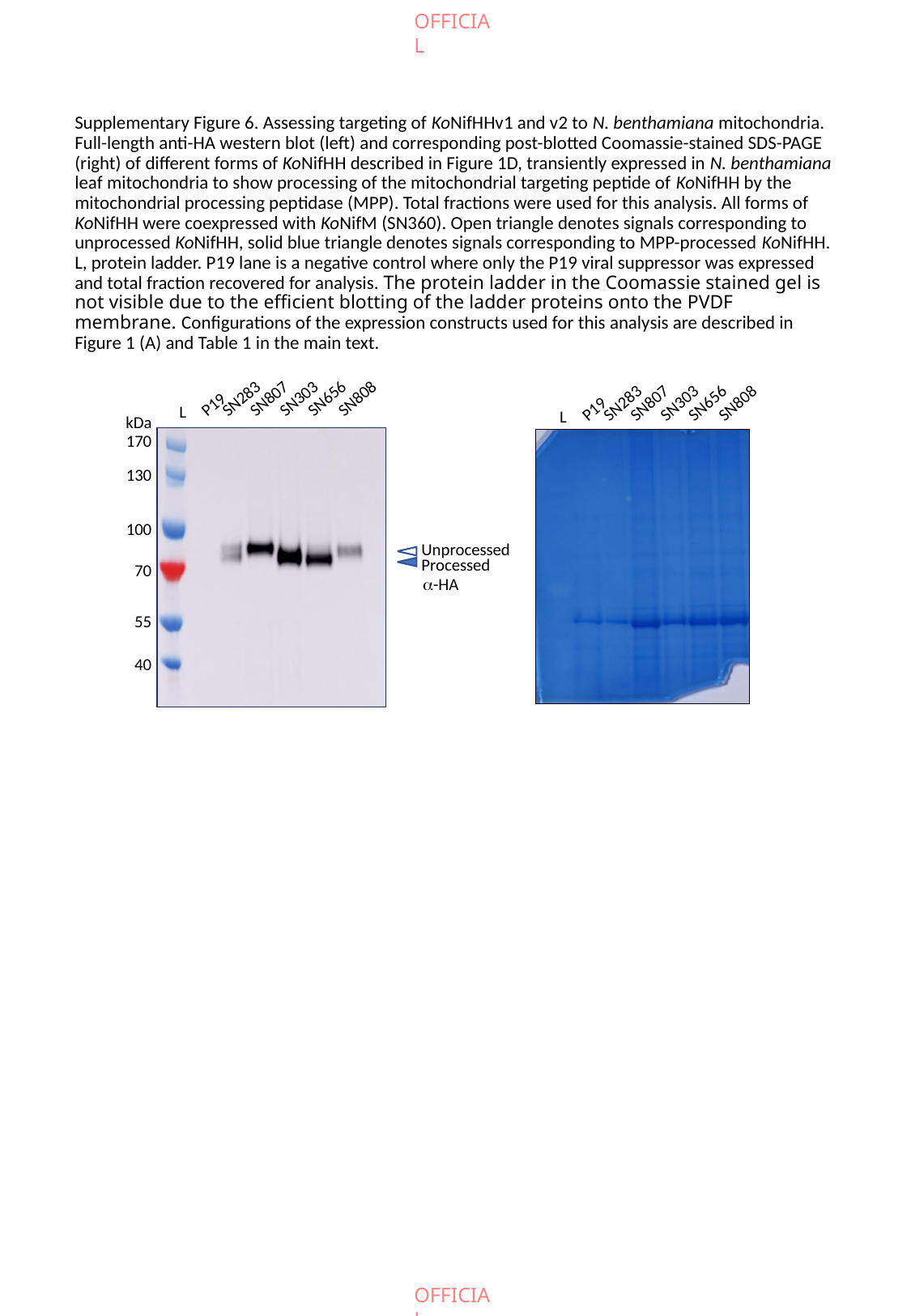

# Supplementary Figure 6. Assessing targeting of KoNifHHv1 and v2 to N. benthamiana mitochondria. Full-length anti-HA western blot (left) and corresponding post-blotted Coomassie-stained SDS-PAGE (right) of different forms of KoNifHH described in Figure 1D, transiently expressed in N. benthamiana leaf mitochondria to show processing of the mitochondrial targeting peptide of KoNifHH by the mitochondrial processing peptidase (MPP). Total fractions were used for this analysis. All forms of KoNifHH were coexpressed with KoNifM (SN360). Open triangle denotes signals corresponding to unprocessed KoNifHH, solid blue triangle denotes signals corresponding to MPP-processed KoNifHH. L, protein ladder. P19 lane is a negative control where only the P19 viral suppressor was expressed and total fraction recovered for analysis. The protein ladder in the Coomassie stained gel is not visible due to the efficient blotting of the ladder proteins onto the PVDF membrane. Configurations of the expression constructs used for this analysis are described in Figure 1 (A) and Table 1 in the main text.
SN808
SN283
SN807
SN303
SN656
P19
L
kDa
170
130
100
70
55
40
SN283
SN807
SN303
SN656
SN808
P19
L
Unprocessed
Processed
α-HA

## Slide 8
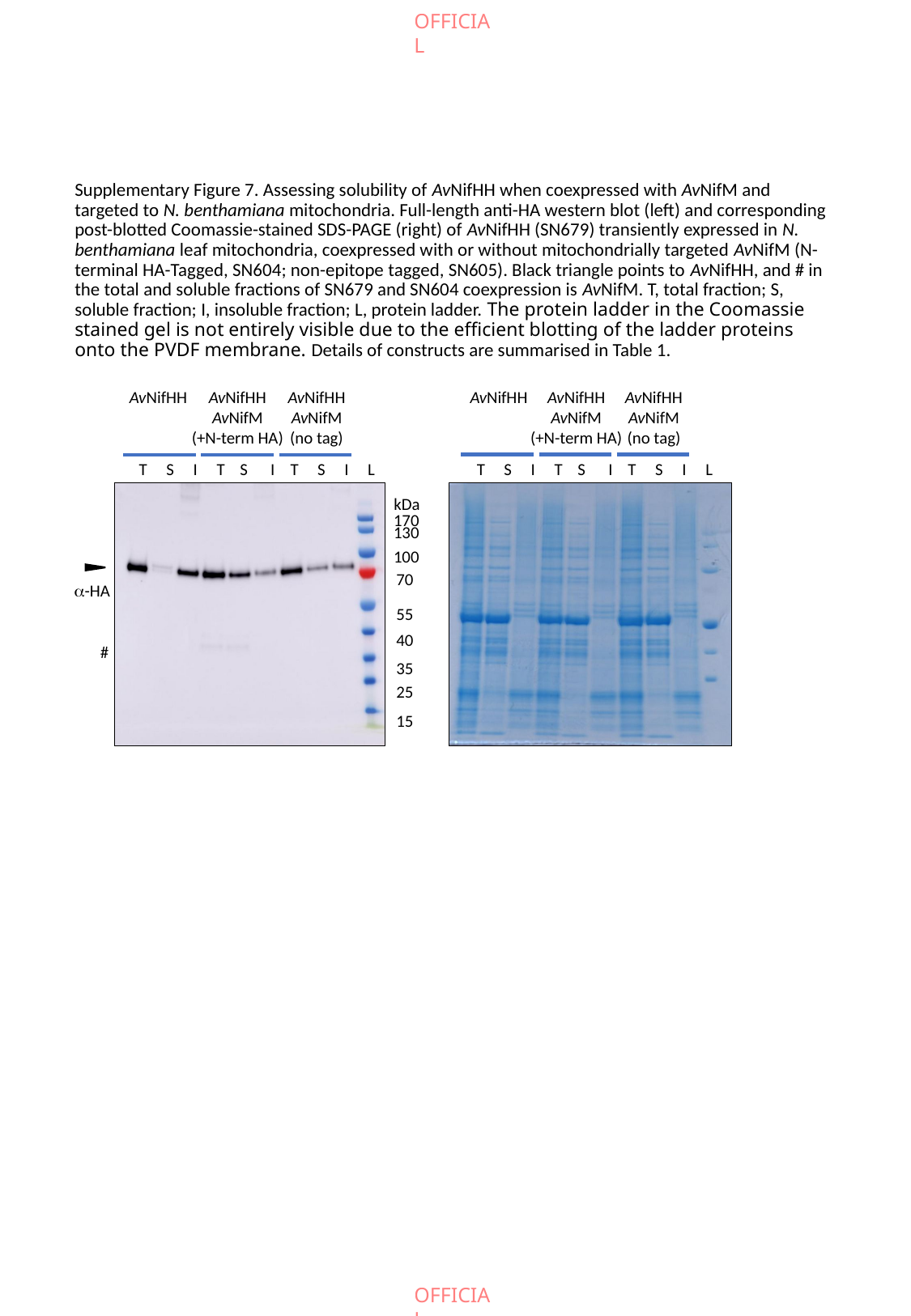

# Supplementary Figure 7. Assessing solubility of AvNifHH when coexpressed with AvNifM and targeted to N. benthamiana mitochondria. Full-length anti-HA western blot (left) and corresponding post-blotted Coomassie-stained SDS-PAGE (right) of AvNifHH (SN679) transiently expressed in N. benthamiana leaf mitochondria, coexpressed with or without mitochondrially targeted AvNifM (N-terminal HA-Tagged, SN604; non-epitope tagged, SN605). Black triangle points to AvNifHH, and # in the total and soluble fractions of SN679 and SN604 coexpression is AvNifM. T, total fraction; S, soluble fraction; I, insoluble fraction; L, protein ladder. The protein ladder in the Coomassie stained gel is not entirely visible due to the efficient blotting of the ladder proteins onto the PVDF membrane. Details of constructs are summarised in Table 1.
AvNifHH
AvNifHH
AvNifM
(+N-term HA)
AvNifHH
AvNifM
(no tag)
T S I T S I T S I L
kDa
170
130
100
70
55
40
35
25
15
α-HA
#
AvNifHH
AvNifHH
AvNifM
(+N-term HA)
AvNifHH
AvNifM
(no tag)
T S I T S I T S I L

## Slide 9
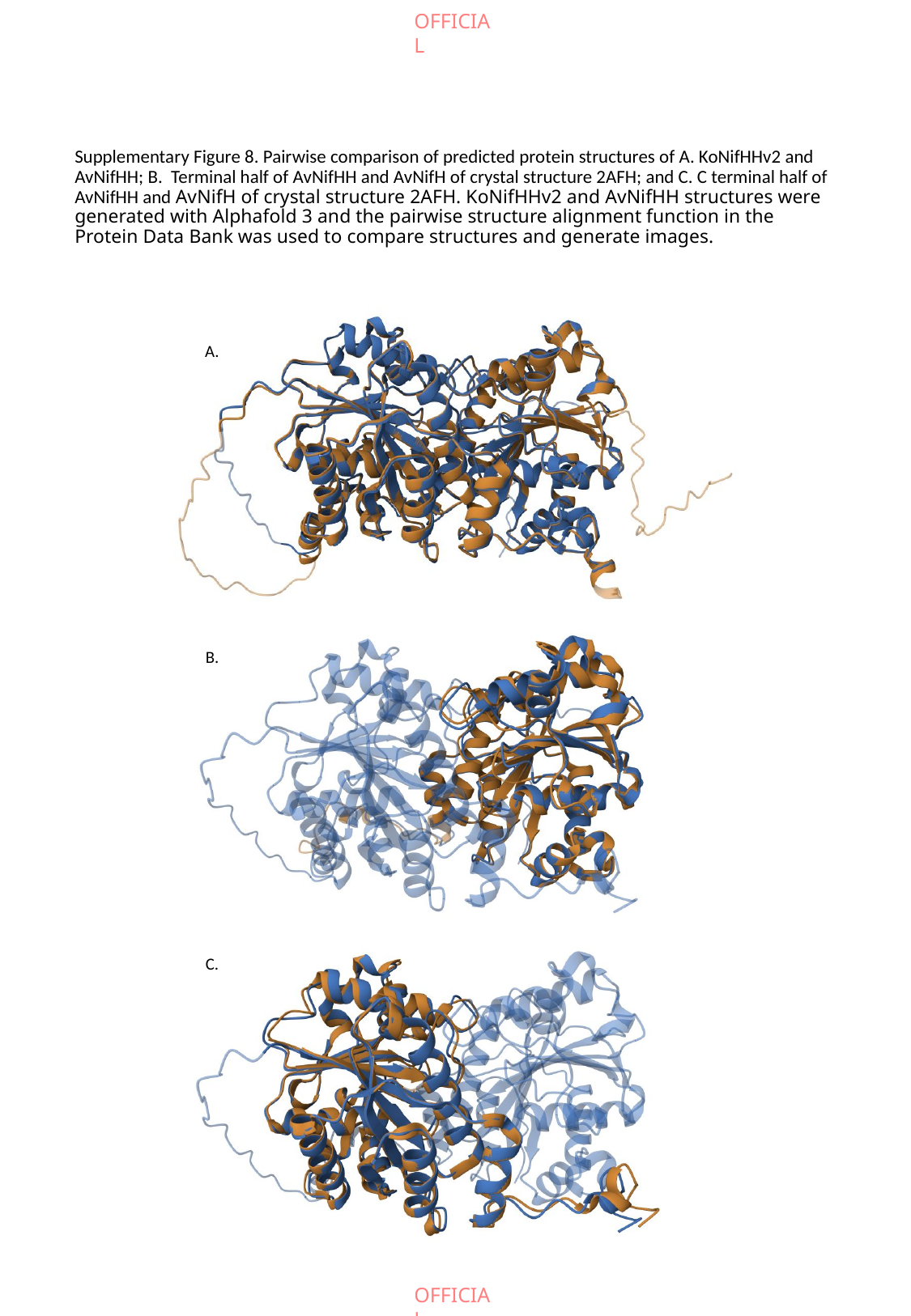

# Supplementary Figure 8. Pairwise comparison of predicted protein structures of A. KoNifHHv2 and AvNifHH; B. Terminal half of AvNifHH and AvNifH of crystal structure 2AFH; and C. C terminal half of AvNifHH and AvNifH of crystal structure 2AFH. KoNifHHv2 and AvNifHH structures were generated with Alphafold 3 and the pairwise structure alignment function in the Protein Data Bank was used to compare structures and generate images.
A.
B.
C.

## Slide 10
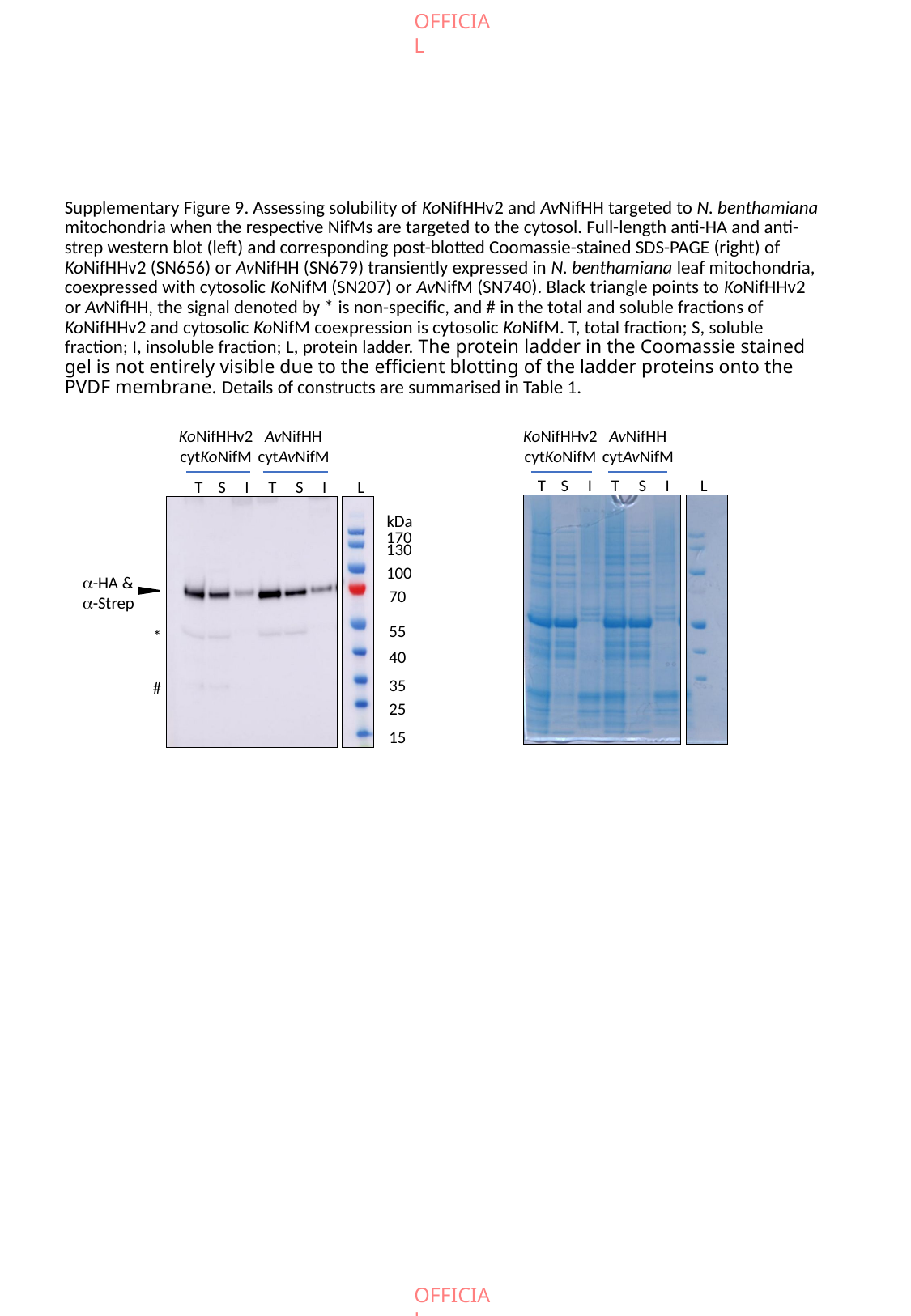

# Supplementary Figure 9. Assessing solubility of KoNifHHv2 and AvNifHH targeted to N. benthamiana mitochondria when the respective NifMs are targeted to the cytosol. Full-length anti-HA and anti-strep western blot (left) and corresponding post-blotted Coomassie-stained SDS-PAGE (right) of KoNifHHv2 (SN656) or AvNifHH (SN679) transiently expressed in N. benthamiana leaf mitochondria, coexpressed with cytosolic KoNifM (SN207) or AvNifM (SN740). Black triangle points to KoNifHHv2 or AvNifHH, the signal denoted by * is non-specific, and # in the total and soluble fractions of KoNifHHv2 and cytosolic KoNifM coexpression is cytosolic KoNifM. T, total fraction; S, soluble fraction; I, insoluble fraction; L, protein ladder. The protein ladder in the Coomassie stained gel is not entirely visible due to the efficient blotting of the ladder proteins onto the PVDF membrane. Details of constructs are summarised in Table 1.
KoNifHHv2
cytKoNifM
AvNifHH
cytAvNifM
T S I T S I L
kDa
170
130
100
70
55
40
35
25
15
α-HA & α-Strep
*
#
KoNifHHv2
cytKoNifM
AvNifHH
cytAvNifM
T S I T S I L

## Slide 11
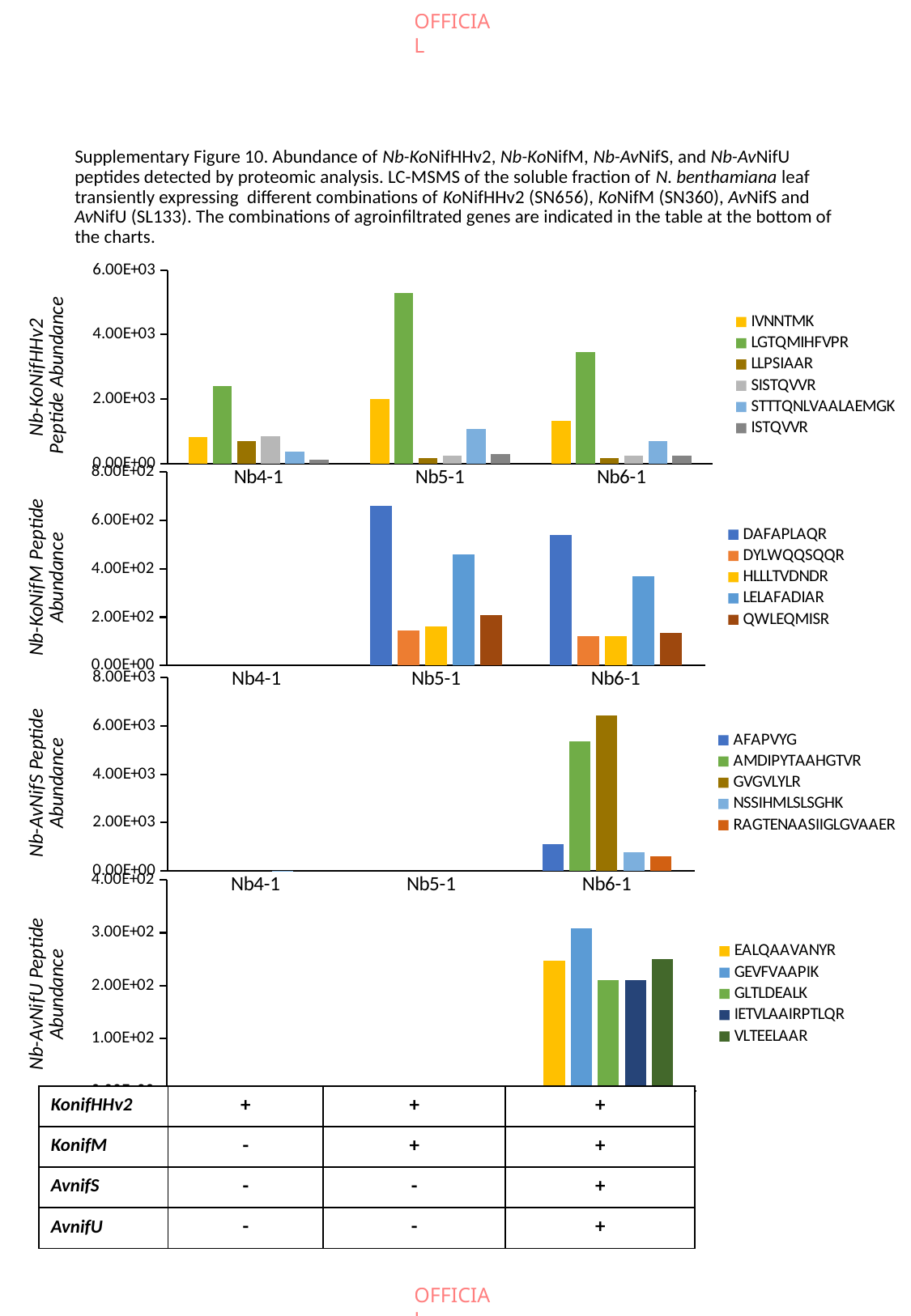

# Supplementary Figure 10. Abundance of Nb-KoNifHHv2, Nb-KoNifM, Nb-AvNifS, and Nb-AvNifU peptides detected by proteomic analysis. LC-MSMS of the soluble fraction of N. benthamiana leaf transiently expressing different combinations of KoNifHHv2 (SN656), KoNifM (SN360), AvNifS and AvNifU (SL133). The combinations of agroinfiltrated genes are indicated in the table at the bottom of the charts.
### Chart
| Category | IVNNTMK | LGTQMIHFVPR | LLPSIAAR | SISTQVVR | STTTQNLVAALAEMGK | ISTQVVR |
|---|---|---|---|---|---|---|
| Nb4-1 | 822.905 | 2400.36 | 702.61 | 835.351 | 353.817 | 119.612 |
| Nb5-1 | 2000.23 | 5284.88 | 163.267 | 237.863 | 1056.55 | 293.939 |
| Nb6-1 | 1308.01 | 3466.52 | 153.896 | 251.438 | 686.561 | 229.642 |
### Chart
| Category | DAFAPLAQR | DYLWQQSQQR | HLLLTVDNDR | LELAFADIAR | QWLEQMISR |
|---|---|---|---|---|---|
| Nb4-1 | None | None | None | None | None |
| Nb5-1 | 660.493 | 146.014 | 161.282 | 458.657 | 207.777 |
| Nb6-1 | 539.119 | 122.118 | 122.361 | 369.489 | 134.697 |
### Chart
| Category | AFAPVYG | AMDIPYTAAHGTVR | GVGVLYLR | NSSIHMLSLSGHK | RAGTENAASIIGLGVAAER |
|---|---|---|---|---|---|
| Nb4-1 | None | None | None | 19.909 | None |
| Nb5-1 | None | None | None | None | None |
| Nb6-1 | 1120.89 | 5363.97 | 6449.52 | 770.44 | 595.591 |
### Chart
| Category | EALQAAVANYR | GEVFVAAPIK | GLTLDEALK | IETVLAAIRPTLQR | VLTEELAAR |
|---|---|---|---|---|---|
| Nb4-1 | None | None | None | None | None |
| Nb5-1 | None | None | None | None | None |
| Nb6-1 | 247.008 | 307.975 | 209.563 | 209.764 | 249.634 || KonifHHv2 | + | + | + |
| --- | --- | --- | --- |
| KonifM | - | + | + |
| AvnifS | - | - | + |
| AvnifU | - | - | + |

## Slide 12
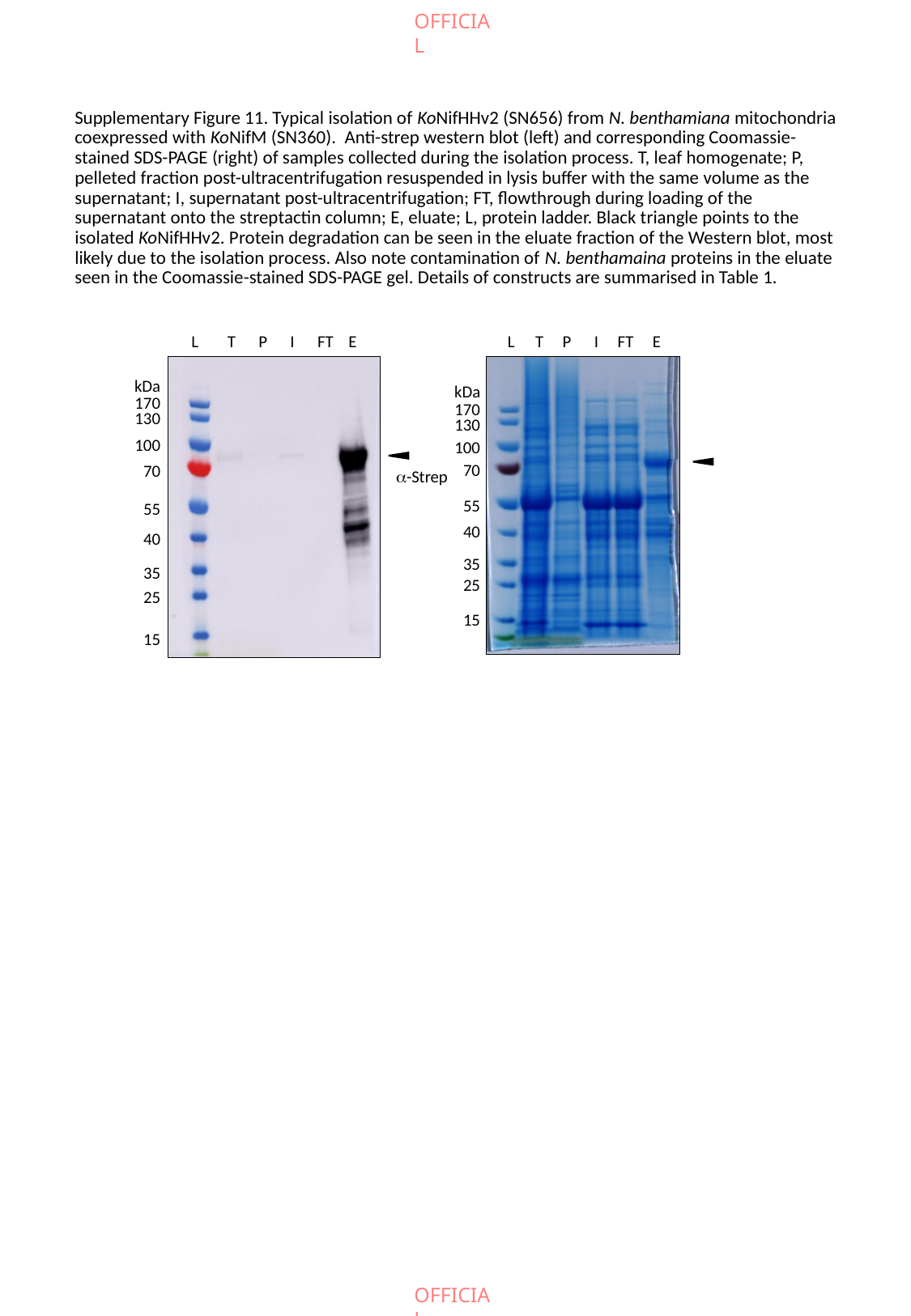

# Supplementary Figure 11. Typical isolation of KoNifHHv2 (SN656) from N. benthamiana mitochondria coexpressed with KoNifM (SN360). Anti-strep western blot (left) and corresponding Coomassie-stained SDS-PAGE (right) of samples collected during the isolation process. T, leaf homogenate; P, pelleted fraction post-ultracentrifugation resuspended in lysis buffer with the same volume as the supernatant; I, supernatant post-ultracentrifugation; FT, flowthrough during loading of the supernatant onto the streptactin column; E, eluate; L, protein ladder. Black triangle points to the isolated KoNifHHv2. Protein degradation can be seen in the eluate fraction of the Western blot, most likely due to the isolation process. Also note contamination of N. benthamaina proteins in the eluate seen in the Coomassie-stained SDS-PAGE gel. Details of constructs are summarised in Table 1.
L
T P I FT E
L
T P I FT E
kDa
170
130
100
70
55
40
35
25
15
kDa
170
130
100
70
55
40
35
25
15
α-Strep

## Slide 13
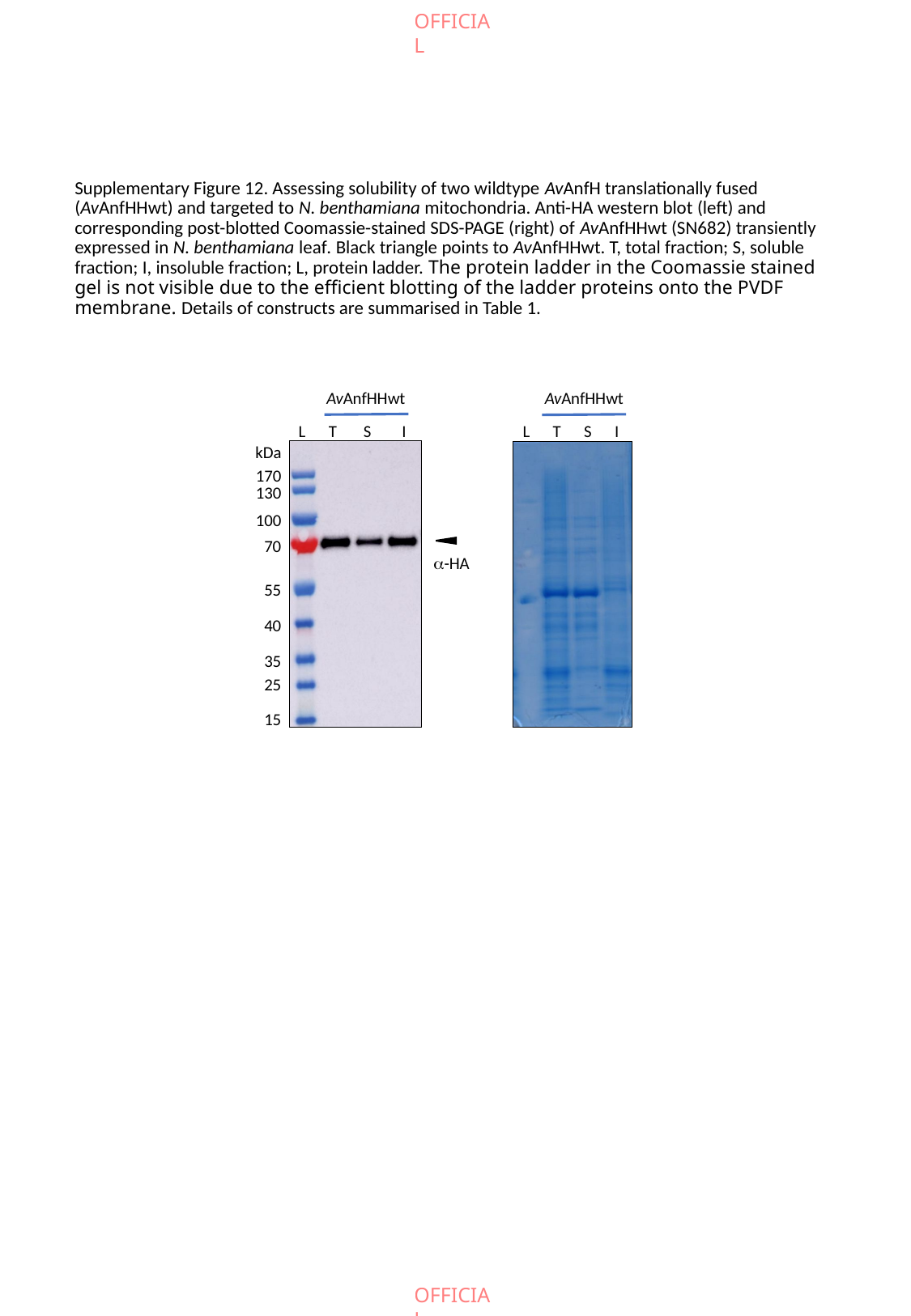

# Supplementary Figure 12. Assessing solubility of two wildtype AvAnfH translationally fused (AvAnfHHwt) and targeted to N. benthamiana mitochondria. Anti-HA western blot (left) and corresponding post-blotted Coomassie-stained SDS-PAGE (right) of AvAnfHHwt (SN682) transiently expressed in N. benthamiana leaf. Black triangle points to AvAnfHHwt. T, total fraction; S, soluble fraction; I, insoluble fraction; L, protein ladder. The protein ladder in the Coomassie stained gel is not visible due to the efficient blotting of the ladder proteins onto the PVDF membrane. Details of constructs are summarised in Table 1.
AvAnfHHwt
L T S I
kDa
170
130
100
70
55
40
35
25
15
α-HA
AvAnfHHwt
L T S I

## Slide 14
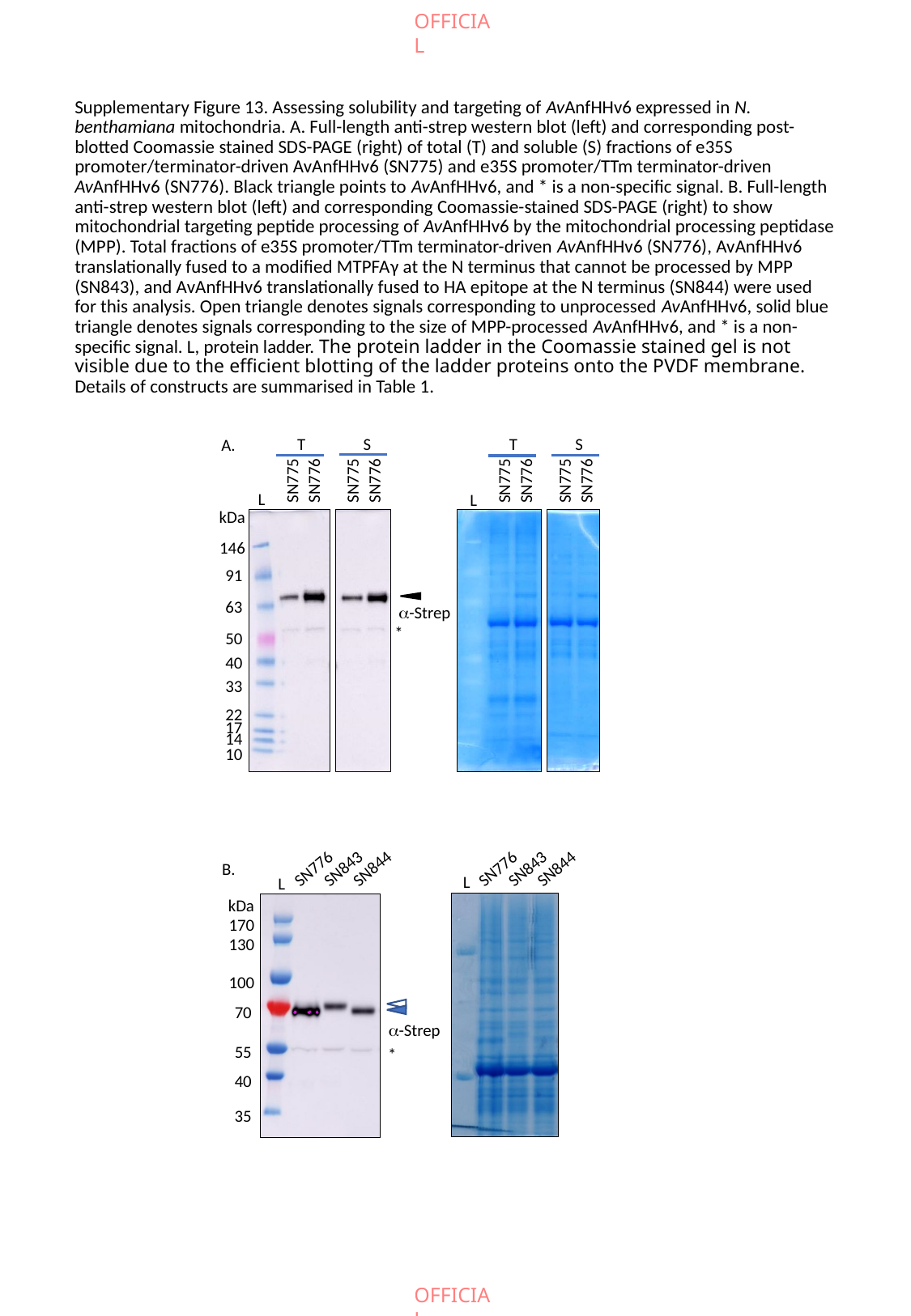

# Supplementary Figure 13. Assessing solubility and targeting of AvAnfHHv6 expressed in N. benthamiana mitochondria. A. Full-length anti-strep western blot (left) and corresponding post-blotted Coomassie stained SDS-PAGE (right) of total (T) and soluble (S) fractions of e35S promoter/terminator-driven AvAnfHHv6 (SN775) and e35S promoter/TTm terminator-driven AvAnfHHv6 (SN776). Black triangle points to AvAnfHHv6, and * is a non-specific signal. B. Full-length anti-strep western blot (left) and corresponding Coomassie-stained SDS-PAGE (right) to show mitochondrial targeting peptide processing of AvAnfHHv6 by the mitochondrial processing peptidase (MPP). Total fractions of e35S promoter/TTm terminator-driven AvAnfHHv6 (SN776), AvAnfHHv6 translationally fused to a modified MTPFAγ at the N terminus that cannot be processed by MPP (SN843), and AvAnfHHv6 translationally fused to HA epitope at the N terminus (SN844) were used for this analysis. Open triangle denotes signals corresponding to unprocessed AvAnfHHv6, solid blue triangle denotes signals corresponding to the size of MPP-processed AvAnfHHv6, and * is a non-specific signal. L, protein ladder. The protein ladder in the Coomassie stained gel is not visible due to the efficient blotting of the ladder proteins onto the PVDF membrane. Details of constructs are summarised in Table 1.
T
S
SN775
SN776
SN775
SN776
L
kDa
146
91
63
50
40
33
22
17
14
10
α-Strep
*
T
S
SN775
SN776
SN775
SN776
L
A.
SN776
SN843
SN844
L
kDa
170
130
100
70
55
40
α-Strep
*
35
SN776
SN843
SN844
L
B.

## Slide 15
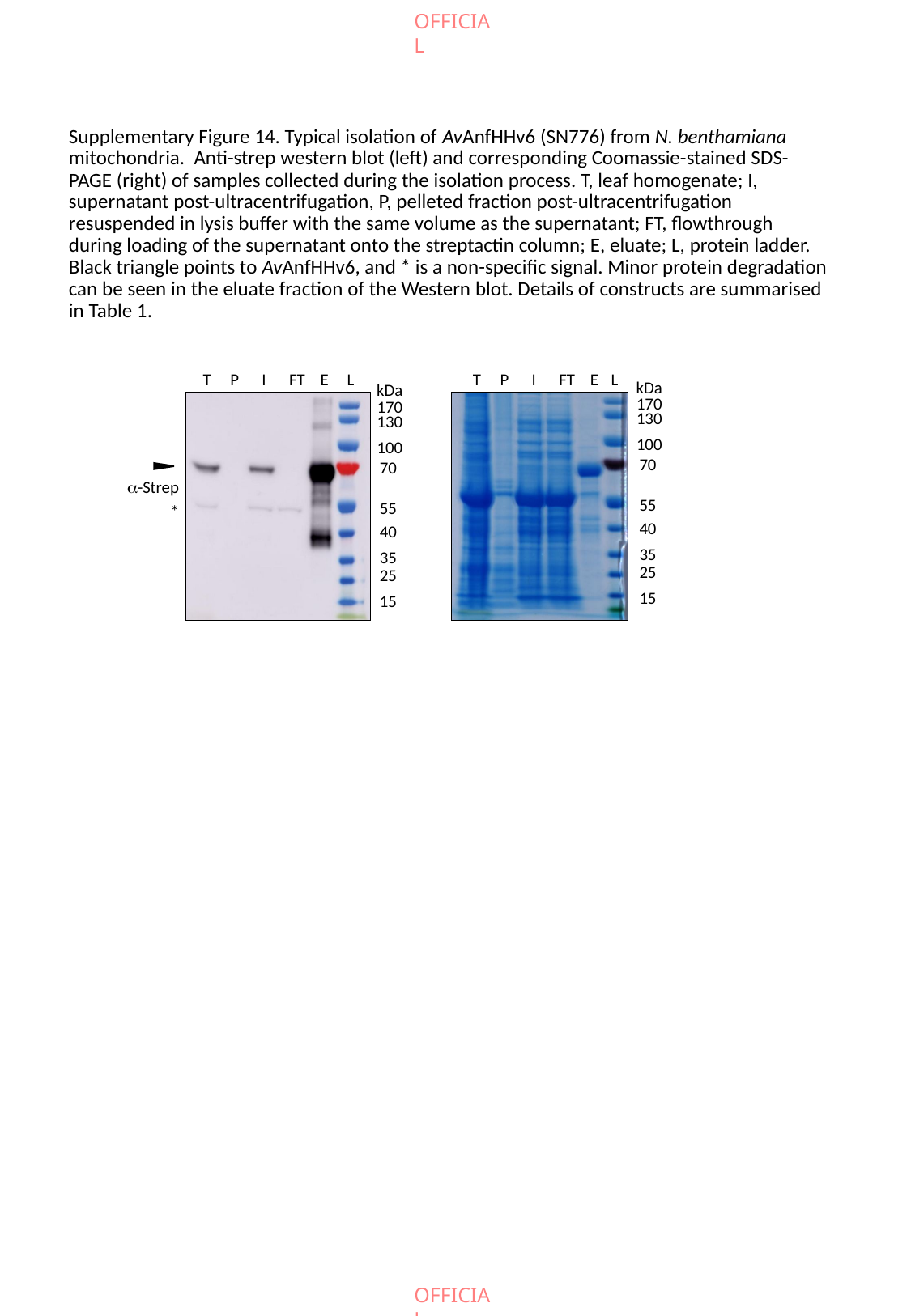

# Supplementary Figure 14. Typical isolation of AvAnfHHv6 (SN776) from N. benthamiana mitochondria. Anti-strep western blot (left) and corresponding Coomassie-stained SDS-PAGE (right) of samples collected during the isolation process. T, leaf homogenate; I, supernatant post-ultracentrifugation, P, pelleted fraction post-ultracentrifugation resuspended in lysis buffer with the same volume as the supernatant; FT, flowthrough during loading of the supernatant onto the streptactin column; E, eluate; L, protein ladder. Black triangle points to AvAnfHHv6, and * is a non-specific signal. Minor protein degradation can be seen in the eluate fraction of the Western blot. Details of constructs are summarised in Table 1.
T P I FT E
L
T P I FT E
L
kDa
170
130
100
70
55
40
35
25
15
kDa
170
130
100
70
55
40
35
25
15
α-Strep
*
